# A Survey on LR-FHSS: State of the Art and Future Directions

**DOI:** 10.3390/s26216843

**Published:** 2026-10-29

**Authors:** Roger Sanchez-Vital, Jana Bodvanski, Lluís Casals, Rafael Vidal, Eduard Garcia-Villegas, Carles Gomez

**Affiliations:** 1 IDLab-Department of Computer Science, University of Antwerp–imec, Sint-Pietersvliet 7, 2000 Antwerp, Belgium; roger.sanchezvital@uantwerpen.be; 2 Department of Network Engineering, Universitat Politècnica de Catalunya, C/Esteve Terradas, 7, 08860 Castelldefels, Spain; jana.bodvanski@gmail.com (J.B.); lluis.casals@upc.edu (L.C.); rafael.vidal@upc.edu (R.V.); eduardo.garcia@upc.edu (E.G.-V.)

**Keywords:** Internet of Things, LoRaWAN, LR-FHSS, survey, state of the art

## Abstract

Long Range–Frequency Hopping Spread Spectrum (LR-FHSS) is a physical layer (PHY) scheme intended to enhance LoRaWAN capabilities in terms of scalability and robustness. Such features qualify LR-FHSS as a promising enabler in direct-to-satellite (DtS) and terrestrial Internet of Things (IoT) environments. Since its publication as part of the LoRaWAN specification, LR-FHSS has attracted increasing attention from the research community. The existing literature has focused on evaluating LR-FHSS performance, often in comparison with former LoRaWAN PHY alternatives (such as LoRa), by means of mathematical analysis, simulation, and real-world experimentation. LR-FHSS performance parameters of interest comprise delivery rate, energy consumption, network capacity, throughput, and latency. The research community has also designed LR-FHSS improvement techniques, either through receiver-side approaches or requiring modifications at both the transmitter and receiver. For the first time, the present paper offers a consolidated view of the state of the art, identifies current challenges, and proposes future directions in the field of LR-FHSS research.

## 1. Introduction

LoRaWAN is a wireless, low-power communication technology intended to provide connectivity for resource-constrained devices, which are typical in Internet of Things (IoT) scenarios. LoRaWAN defines a star-of-stars network topology, where end-devices (EDs) communicate with a network server (NS) via nearby gateways (GWs). In LoRaWAN, physical layer (PHY) transmission between EDs and GWs has primarily been based on the modulation called LoRa [1].

Like other low-power wide-area network (LPWAN) technologies, LoRa/LoRaWAN offers a long link range, in the order of up to several kilometers, while allowing multiyear lifetime for battery-operated devices [1,2]. Thanks to its open approach, flexibility, and performance, LoRaWAN has become a popular technology, with forecasts predicting the number of LoRa/LoRaWAN devices exceeding 1 billion by 2030 [3].

Since the inception of LoRaWAN, one of its most significant enhancements has been the introduction of a new PHY scheme called long-range frequency-hopping spread spectrum (LR-FHSS) [4]. Motivated by the high momentum of satellite-based communication, LR-FHSS is intended to support direct-to-satellite (DtS) IoT use cases. Such environments demand increased scalability and robustness, as a single LoRaWAN GW carried by a low Earth orbit (LEO) satellite may need to serve thousands of EDs under its coverage [5].

**Figure 1:**
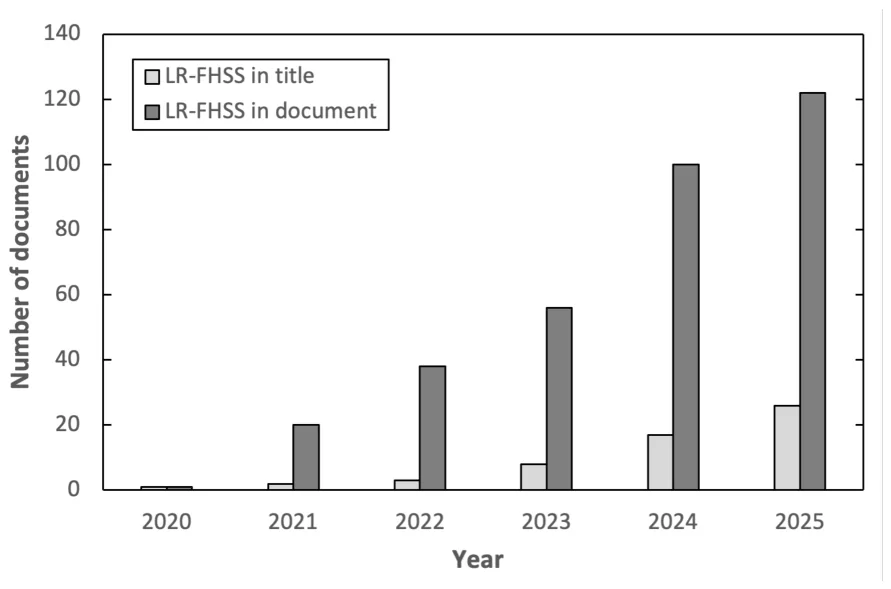
Number of published documents including “LR-FHSS” in their title or within the document, up to and including 2025 (Source: Google Scholar).

Since its introduction in 2020, LR-FHSS has attracted growing attention from the research community (cf. Figure 1). However, as of the writing, there is no published work offering a consolidated view of the knowledge gained and open issues in the LR-FHSS field. In order to fill this gap, the present paper provides a survey on LR-FHSS, which explores the related literature up to the 1st of October 2026 [6,7,8,9,10,11,12,13,14,15,16,17,18,19,20,21,22,23,24,25,26,27,28,29,30,31,32,33,34,35,36,37,38,39,40,41,42,43,44,45,46,47,48]. The survey comprises the following works, as found by Google Scholar: (i) all publicly available research works written in English containing the keyword ”LR-FHSS” as part of their title, yielding 43 entries, and (ii) works that include the term “LR-FHSS” in the document but not in the title, yet provide a significant contribution in terms of LR-FHSS research, adding 2 further entries. Thus, this survey covers in detail a total of 45 entries on LR-FHSS. Note that, in order to avoid redundancy, early presentations of results that have been included in subsequently published works have been excluded from this survey.

The remainder of this paper is organized as follows. Section 2 describes the fundamentals of LoRaWAN and LR-FHSS. Section 3, Section 4, Section 5 and Section 6 provide a structured overview of LR-FHSS research, in the areas of evaluation tools (Section 3), performance (Section 4), proposed improvement techniques (Section 5), and applications (Section 6). Section 7 highlights the main current challenges and future research directions in LR-FHSS. Finally, Section 8 concludes this paper.

## 2. LoRaWAN and LR-FHSS: An Overview

The LoRaWAN specification provides both the system architecture and the communication protocol for this LPWAN technology [49]. At its core, the architecture relies on a triad of components: EDs, GWs, and a centralized NS.

**Figure 2:**
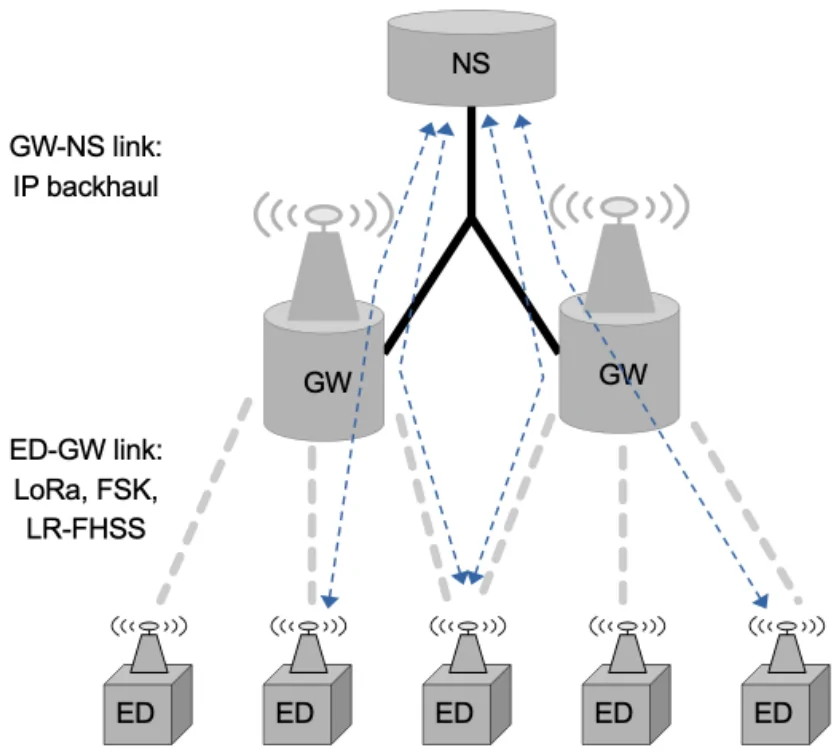
LoRaWAN network architecture.

The basis is a star topology, in which communication naturally flows from the edge to the core (cf. Figure 2). EDs transmit data to the NS via one or multiple GWs. While these GWs bridge to the NS using standard, high-bandwidth Internet Protocol backhauls (e.g., Ethernet, 4G, or 5G), the EDs rely on specialized wireless links. Historically, these wireless links have utilized LoRa or frequency-shift keying (FSK) modulations, but the ecosystem has recently expanded to include LR-FHSS.

### 2.1. LoRa Modulation Characteristics

Standard LoRa modulation is based on the chirp spread spectrum (CSS) technique [50]. In this approach, a radio frequency signal sweeps from the lower limit to the upper limit of a designated channel bandwidth (typically 125 kHz, 250 kHz, or 500 kHz), depending on regional regulations [51].

LoRa is widely used in IoT deployments because of its robustness to noise and interference, very long communication range, and very low power consumption. It uses orthogonal spreading factors (SFs), which allow many devices to share the same frequency channel efficiently. Higher SFs improve reliability but reduce the data rate, as shown in Table 1. Because LoRa provides a higher processing gain than many other modulation schemes, devices can transmit using less power. In addition, LoRa incorporates error-correction mechanisms that help make transmissions more robust and reliable.

### 2.2. The Evolution with LR-FHSS

Semtech introduced LR-FHSS to further improve network scalability and resilience to interference [6,25]. Moving away from CSS, LR-FHSS employs rapid frequency hopping across ultra-narrow sub-carriers. This design reduces signal collisions, allowing a great number of devices to transmit uplink messages simultaneously. Currently, LR-FHSS is applied exclusively to uplink traffic, while downlink messages continue to rely on LoRa.

**Figure 3:**
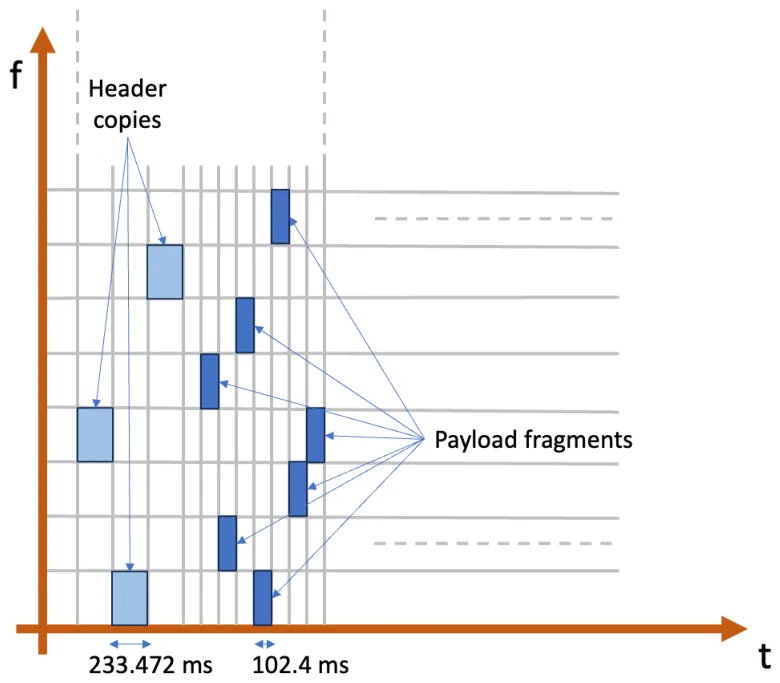
LR-FHSS transmission procedure.

Transmitting with LR-FHSS requires a highly orchestrated, redundant process. When a packet is sent, the PHY header is replicated two or three times (depending on the data rate (DR)), with each duplicate hopping to a new sub-carrier aiming to enhance delivery rate (cf. Figure 3). The transmission of this header takes precisely 233.472 ms. The payload that follows is sliced into smaller fragments of 102.4 ms, with each chunk transmitted on a different sub-carrier. These elements are sent using Gaussian minimum-shift keying (GMSK) modulation, where one symbol equals one bit [31]. Convolutional coding is used for error correction, with coding rates (CRs) of 1/3 or 2/3.

### 2.3. Regional Parameters and Payload Capacities

Network configurations and transmission time limits are defined by the LoRa Alliance’s Regional Parameters specification [51]. Because LPWANs operate in unlicensed spectrum, they must strictly adhere to local regulations, which indicate how LR-FHSS is utilized in different regions, such as Europe (EU) or the United States (US).

#### 2.3.1. The European Context (EU 863–870 MHz Band)

In Europe, the LoRaWAN specification for the 863–870 MHz band outlines specific DR profiles across LoRa, FSK, and LR-FHSS (summarized in Table 1). N/A denotes “not applicable” in the tables.

**Figure 4:**
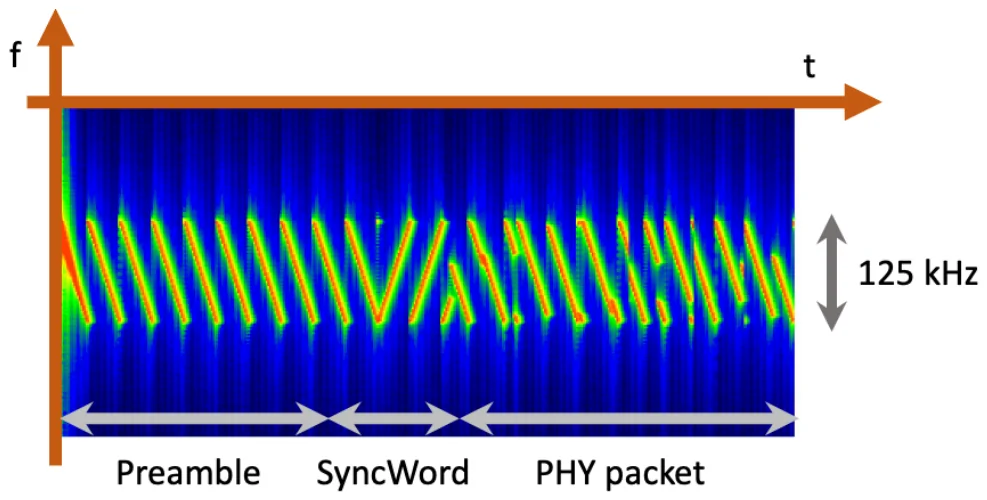
Time-frequency capture of a LoRa signal.

For DRs DR0 through DR5, LoRa uses SFs ranging from SF12 down to SF7, respectively, with a bandwidth of 125 kHz; while DR6 uses SF7 with a 250 kHz bandwidth. Figure 4 illustrates a real LoRa signal, containing up-chirps and down-chirps. Higher SFs result in lower bit rates that provide greater transmission reliability and robustness. With these settings, the achievable bit rates range from 250 bps up to 11 kbps. DR7 switches to FSK modulation, which supports a much higher bit rate of 50 kbps. In its latest Regional Parameters specification [51], the LoRa Alliance introduces two additional DRs, DR12 and DR13. Based on LoRa and using a new SF, DR13 can achieve DRs of up to 15,625 bits per second. It is worth noting that EDs are only required to support DR0 through DR5.

The more recent LR-FHSS modulation is used in DRs DR8 through DR11. LR-FHSS integrates a 488 Hz per-hop bandwidth alongside two distinct CRs (1/3 and 2/3) and operates across channel widths of either 137 kHz or 336 kHz. Consequently, these combined settings yield two available bit rate choices: 162 bps and 325 bps.

LoRa and LR-FHSS use different frame structures, and their maximum medium access control (MAC) payload size changes depending on the specific DR utilized. For instance, while DR0 accommodates up to 51 bytes, DR8 and DR9 are slightly lower, with a limit of 50 bytes. Higher capacities are seen with DR10 and DR11, which can handle up to 115 bytes, but the absolute highest payload limit belongs to DR7, which is capable of carrying 242 bytes.

#### 2.3.2. The US Context: 902–928 MHz Band

In the US, Federal Communications Commission (FCC) regulations enforce a strict 400-millisecond dwell-time limit per channel. For standard LoRa, this means devices cannot legally use SFs higher than SF10. As a result, standard LoRa payloads are capped at 11 bytes for DR0, and the maximum network range is inherently restricted since the robust SF11 and SF12 configurations cannot be used.

LR-FHSS conforms to these regulations. By rapidly hopping across a wide 1.523 MHz operating bandwidth (with at least 25.4 kHz spacing between hops), an LR-FHSS transmission never stays on a single sub-channel long enough to violate the 400 ms rule. Specified for the US DR5 and DR6 (cf. Table 2), LR-FHSS allows devices to send payloads of 50 bytes and 125 bytes, respectively, while effectively matching the long-range link budgets of LoRa’s SF11 and SF12.

Performance-wise, US DR5 and DR6 are similar to EU DR8-DR10 and EU DR9-DR11, respectively. In the remainder of this paper, we refer by default to EU DRs, otherwise stating explicitly whether DRs correspond to another region.

### 2.4. Device Classes and Network Activation

LoRaWAN categorizes EDs into three distinct operational classes: A, B, and C. Class A is the universal, mandatory, and most energy-efficient mode. It utilizes an ALOHA-based transmission protocol where the ED is in control of the communication; the NS can only transmit a downlink message during one of two reception windows that open shortly after an uplink message. Transmissions can be sent as unconfirmed messages or confirmed messages, the latter requiring an acknowledgment (ACK) from the NS.

Before any ED can participate in the network, it must undergo activation. The specification provides two activation pathways: activation by personalization (ABP) and over-the-air activation (OTAA). ABP involves hardcoding the necessary parameters into the device prior to field deployment. Conversely, OTAA is a dynamic, over-the-air handshake.

It begins with the device broadcasting a Join-Request and waiting for a *Join-Accept* from the NS. Once successfully joined, the device is securely provisioned with a network identifier, an address, encryption keys, and specific radio configurations, such as preferred channels, transmission power, and target DRs, among others.

## 3. State of the Art: LR-FHSS Evaluation Tools

This section reviews the tools developed and/or used by researchers in order to evaluate the performance of LR-FHSS. The majority of LR-FHSS evaluation works combine analytical modeling with validation by means of simulation, whereas a few studies are based on experiments carried out in real-world testbeds. Table 3 features the list of all surveyed documents. The methods used to evaluate each LR-FHSS improvement technique reviewed in this survey, along with corresponding outcomes and applications, can be found in Table 12.

### 3.1. Analytical Models

Most of the reviewed works examine LR-FHSS performance by using analytical models. These models are generally used to evaluate link reliability and energy performance. Link performance models, which are used to explore signal detection, interference handling, and coverage, are discussed in [7,10,12,13,14,18,19,20,21,22,23,26,28,30,31,34,37,39,41,43,46,48]. Conversely, models focusing on power usage and battery lifetime are found in [8,15], and [27]. Bez Rocha et al. in [40] consider both link and energy models, whereas Fraire et al. in [21] address the headerless frame recovery problem. The specifics of these models are indicated in Table 4.

#### 3.1.1. Link Models

A variety of models have been developed for LR-FHSS link performance evaluation. These models can be broadly categorized into satellite communication models, receiver and interference models, resource allocation models, and channel access models.

A significant portion of the literature focuses on modeling LR-FHSS in satellite and DtS environments. System, propagation, collision, and capture effect models have been utilized to evaluate packet loss specifically for DR8 and DR9, validated through simulations [7]. These models were later extended to evaluate frame and fragment replication techniques aimed at increasing message delivery probability [22]. The latter improvements come at the cost of increased energy expenditure, so the authors also include an energy-efficiency model. Packet loss rate has also been analytically determined under various frame header placement modification approaches, in order to minimize the impact of correlated interference on repeated frame headers [45]. Correlated errors arriving in bursts or clusters are the focus of [52], where the authors develop analytical models for segment collisions, packet loss, and energy consumption under Poisson traffic, pure ALOHA access, and a shadowed-Rician satellite channel. Although they retain the header/payload structure, its numerical evaluation assumes binary phase-shift keying (BPSK) rather than GMSK, limiting direct comparison with standard LR-FHSS.

Outage probability in satellite links is another major focus. Analytical models in this area have accounted for noise, disconnection probability due to fading, the capture effect, and ground-to-satellite path loss to assess transmission reliability [10]. Similar models have been applied to D2D-aided satellite IoT networks under shadowed-Rice fading channels, demonstrating how relay nodes can improve link reliability for devices lacking direct satellite visibility [13]. Sensitivity-based outage is also modeled for LEO satellite and high-altitude platform station (HAPS) links using a rural non-terrestrial channel model [41]. Similarly, the DEWASAT-1 case study also compares LoRa-CSS and LR-FHSS through link-margin and airtime calculations [43].

At the PHY, transceiver architectures for LEO satellites have been detailed with mathematical formulations for Doppler shift estimation and compensation, signal detection, and synchronization in dynamic satellite channels [14]. These formulations have been further utilized to represent LEO satellite channel impairments and define signal processing algorithms for the proposed LR-FHSS transceiver [18]. Other PHY models for DtS-IoT environments observe how signals behave in LR-FHSS transmission, considering real-world effects [20]. To keep the models both simple and realistic, they assume that there are no small-scale fading or atmospheric losses, including Doppler effects, and rely on robust collision detection methods. It is also important to note the practical assumption that fragment collisions occur when signals overlap in time and frequency and that no recovery is possible. Additionally, the authors in [26] provided models to evaluate the reliability of long-range LPWAN communication systems. Key parameters, such as ToA, were incorporated to assess latency and identify potential issues like delays, packet errors, or packet loss.

Modeling signal detection and interference at the GW is critical for enabling massive IoT connectivity. Mathematical models have been proposed for receivers capable of simultaneous signal reception, analyzing how interference blanking techniques can decode overlapping signals to mitigate collisions [12]. At the modulation level, models of the GMSK transmitter and channel have facilitated the development of a low-complexity GMSK signal detection scheme at the GW, proving more efficient than the traditional Viterbi algorithm [19]. To establish theoretical capacity limits, analytical models have also evaluated LR-FHSS networks utilizing successive interference cancellation (SIC); by iteratively calculating expected interference and decoding success probabilities (differentiating between the original power of lower-power packets and the residual power of higher-power packets), these models reveal the theoretical capacity limit of the network [39].

Optimizing how LR-FHSS structures and distributes its transmissions has led to various predictive and allocation models. A packet reception rate (PRR) prediction model was developed for dynamic frequency hopping (DFH) based on per-hop channel quality and the effects of frequency-hopping sequences on decoding success [46]. To maximize goodput, analytical models have been used to predict the optimal number of header replicas based on payload size and CR [31]. The authors considered more detailed assumptions than the prior literature, such as losses due to interference produced by neighboring subchannels.

Broader system success probabilities have been modeled alongside optimization strategies that allocate header and payload channels based on payload size, DRs, and the number of devices in the network [28]. Furthermore, the authors in [23] mathematically modeled how the transmission setup affects the number of headers and payload fragments sent per second, the collision risks during vulnerable periods, and how they affect message delivery success. These models were used to find the best way to assign setup probabilities to devices, which helps the network achieve the highest possible goodput and energy efficiency. Packet success probability has also been modeled to explore the benefits of macro-diversity [34]. In their model, packet success probability relies on two independent conditions: (i) at least one GW successfully decodes a header replica; and (ii) a sufficient number of payload fragments are collectively received across the GWs and forwarded to the network controller.

Regarding channel access, a robust MC^2^AMB model has been proposed to determine the optimal packet lengths, number of devices, and header replicas required to maximize packet success probability [37]. The authors of [48] also model the average success delivery ratio and goodput of an LR-FHSS probabilistic header repetition mechanism combined with SIC. Similarly, the authors in [40] propose an analytical model to evaluate the performance of three message transmission techniques for LR-FHSS: concatenation, embedded retransmission, and replication. This model incorporates an on-off fading model to account for channel impairments, representing scenarios where packet losses are dominated by fading and short-term blocking. Since the network employs ALOHA-based multiple access, the model also accounts for destructive collisions that lead to lost headers or payload fragments. Using this framework, the analytical model estimates several key performance metrics, including the overall message success probability and the average and maximum transmission delays for each technique. Finally, moving beyond theoretical analysis, simple empirical path-loss models have been developed using linear regression coefficients and transmitter–receiver distance data gathered from real-world urban measurement campaigns [30].

#### 3.1.2. Energy Models

Several recent studies have focused on characterizing the LR-FHSS energy consumption from various perspectives. For instance, a comprehensive analytical model was developed to evaluate critical metrics such as average ED current consumption, battery lifetime, and the energy cost per data transmission [8]. This framework specifically benchmarks the energy efficiency of LR-FHSS against the legacy LoRa modulation across different DRs, based on real hardware measurements. Other researchers have also grounded their energy models with experimental measurements, characterizing the current consumption and ToA of real hardware to provide realistic battery life estimations for LR-FHSS EDs [15].

Beyond single-device evaluations, energy consumption has also been analyzed from a broader topological standpoint. A recent analysis of a two-tier network architecture compares the energy cost of a direct sensor-to-satellite link against a relayed setup, where sensors use LoRa to reach a GW that subsequently backhauls the data to a satellite via LR-FHSS [27].

Finally, even in studies where energy modeling is not the primary focus, power consumption remains a vital performance indicator; for example, recent work proposing new transmission techniques to improve network scalability also calculated the average energy consumption per successfully delivered message to comprehensively evaluate their proposed methods [40].

#### 3.1.3. Other Models

The authors in [21] formulated an integer linear programming (ILP) model to address the headerless frame recovery problem. The model defines the constraints for reconstructing fragmented messages based on payload content when physical headers are lost due to interference. Compound interference recognition is also formulated as multi-instance–multi-label learning, with each signal sample labeled by its active interferers [47].

Joint LoRa/LR-FHSS access is benchmarked using 2-D and sequential 1-D LoRa scheduling models with link-budget and Doppler constraints [42].

### 3.2. Simulators

A set of studies rely on simulation tools, as observed in [6,7,9,16,18,19,20,21,22,23,26,27,28,31,35,36,37,38,40,41,42,46,47,48], many of which are custom-built using Python or MATLAB. In other cases, details about the simulation tool used are not provided. An overview of the simulators is presented in Table 5.

#### 3.2.1. Python

To evaluate the performance of LR-FHSS, researchers have predominantly relied on Python-based simulation frameworks, ranging from custom discrete-event scripts to comprehensive open-source tools. Early studies developed tailored packet-based simulators to be able to analyze and compare the performance of LoRa with that of LR-FHSS [6]. Others implemented a discrete-event simulator within the SciPy framework in Python [23]. Providing a more granular look at the PHY, the authors in [31] validated and tested their provided analytical models via a discrete-event simulator implemented in Python. This simulator captures the operation of an LR-FHSS with many EDs and one GW. Packet headers and payload fragments are sent by using subchannels, following the FHS. Then, the simulator determines frame overlaps. A header replica is correctly received if less than 50% of its encoded bits collide, and no collisions affect the synchronization sequence. For an a/b CR, a payload fragment is assumed to be correctly received if no more than 8L(b−a) bits suffer collisions.

As the need for standardized evaluation grew, many tools drew inspiration from established LoRa frameworks. For instance, an early LoRaSim-inspired simulator was adapted to test the throughput and energy efficiency of LR-FHSS employing ACRDA techniques [9]. Formalizing this approach, ref. [16] introduced LR-FHSS-Sim, an open-source, SimPy-based discrete-event simulator. By significantly improving upon LoRaSim’s software design and documentation, LR-FHSS-Sim efficiently models packet transmissions, interference, and network behavior. Consequently, it has become a basic tool in the recent literature. Researchers have leveraged LR-FHSS-Sim to evaluate their orthogonal PHY channels proposal [28], evaluate probabilistic header repetition with SIC [48], benchmark new models against standard LR-FHSS and the state-of-the-art balls-into-bins model (BBM) [7,37], and evaluate the proposed transmission techniques under varying network conditions, such as different traffic loads, background traffic levels, and high network densities of up to 50,000 EDs [40]. By incorporating the proposed techniques to measure message success probability, delay, network congestion, and energy consumption, this study provides practical insights into the inherent trade-offs of each method.

Beyond general-purpose tools, several studies have developed tailor-made SimPy environments to address specific use cases. For example, the authors in [18] verified their proposed transceiver mechanisms using the custom-made LoRa-Space simulation tool. In optimization-focused research, tailored Python simulators have been integrated with the Gurobi optimizer to solve ILP problems associated with headerless frame recovery [21]. This specialized setup was later enhanced to simulate complex spatial and terrestrial scenarios [20]. Joint-access simulations use Skyfield/SGP4 for orbit-aware multi-satellite evaluation [42].

Finally, to push the boundaries of network capacity, comprehensive simulation campaigns have been designed to test scalability and advanced decoding enhancements. Most notably, the authors in [38] conducted an extensive simulation campaign to evaluate the scalability and decoding performance of their proposed LR-FHSS enhancements. The simulations were configured using parameters for DR8 and DR9, modeling a single digital signal processor GW with a 200 kHz channel spacing. To assess network capacity under varying traffic loads, the primary control parameter was the number of nodes, which scaled from 10 to 10,000 devices, each transmitting a single payload during the simulation. Throughout these tests, the authors evaluated their newly proposed FHSs (like the Li-Fan wide-gap sequence) alongside dynamically toggled demodulator allocation strategies (“Early Decode” and “Early Drop”). Additionally, they scrutinized mechanisms like “Early Header Drop” and “Header Tolerance” to observe how header collisions and missing data impacted overall payload recovery.

#### 3.2.2. MATLAB

In addition to Python frameworks, MATLAB has also been adopted for LR-FHSS evaluation, particularly for Monte Carlo simulations and detailed PHY modeling. Several studies utilize MATLAB-based Monte Carlo simulators to validate theoretical predictions against simulated network behaviors. For instance, researchers have verified analytical models for DtS scenarios, testing packet loss across varying distances and node densities for DR8 and DR9 [7]. Similar Monte Carlo models have been used to evaluate satellite-based communications [26], exploring message replication reliability [22]. In [46], Monte Carlo simulations are used to predict PRR for candidate frequency-hopping sequences, with prediction accuracy validated against real-world measurements.

MATLAB is also highly effective for trace-driven PHY simulation. To evaluate a novel SIC method, the authors in [39] developed a software receiver setup that introduces real-world packet signals into a Long-Term Evolution (LTE) extended typical urban (ETU) channel model. This approach successfully replicates challenging physical environments characterized by multipath fading and large delay spreads. The simulation generates 10 s signal traces starting as unit-power noise, into which randomly timed, frequency-hopped packets are injected. These packets are scaled to randomly selected signal-to-noise ratio (SNR) values ranging from −17 to 3 dB for DR8 and −13 to 7 dB for DR9. By employing a two-antenna receiver configuration, this demanding environment tests the network’s capacity limits under heavy traffic loads of up to 1000 packets (approximately 9.6 kbps).

#### 3.2.3. Other Simulation Approaches

The authors in [19,33] developed a tailor-made simulator to test the performance of their low-complexity detection proposals in terms of BER. In [19], a quadrature-based transmitter and shadowed-Rice fading channel were used to simulate ground-to-satellite conditions. In [33], additive white Gaussian noise (AWGN) and Rayleigh fading channel models are used to simulate the interference a terrestrial LoRa GW experiences from an LR-FHSS DtS transmitter. Synthetic LR-FHSS signals with shadowed-Rician fading, time-varying Doppler, and up to three simultaneous interferers are used to evaluate recognition methods in [47].

It is also worth noting the approach in [35], where the authors do not disclose specific details of the simulation platform, yet they ensure a highly accurate, standard-compliant evaluation by directly leveraging Semtech’s SWDM001 LR-FHSS software drivers to build their models. Also omitting specific framework details, the work in [36] evaluates the performance of LR-FHSS by simulating the entire physical and link-layer sequence (from GMSK modulation to receiver decoding) to obtain metrics like BER and ToA under different SNR and CR configurations. A hybrid methodology combining real measurements and simulation is followed in [46]. The authors collect traces of real LR-FHSS packets and interference profiles and then combine them to simulate signals under various interference conditions. Trace-driven simulation is also used in the same work to emulate the concurrent transmissions by a large number of nodes. Monte Carlo simulation is also used to evaluate outage in LEO/HAPS links [41].

### 3.3. Testbeds

Several studies have evaluated LR-FHSS performance through empirical measurements (cf. Table 6), providing valuable information about LR-FHSS operation under practical deployment conditions, such as interference, mobility, and high-altitude communication. These include communication functions implemented using application-specific integrated circuits (ASICs) and field-programmable gate arrays (FPGAs) [18]; out-of-the-box commercial devices [8,15,24,25,29,30,32,43]; and a mix of both software-defined and commercial devices [17,46].

First, the authors in [18] implemented a proposed LR-FHSS transceiver design and synchronization algorithm using an ASIC-based transmitter and an FPGA-based receiver to assess its performance under real-world conditions.

Other researchers have focused on comprehensive performance profiling using physical devices. Laboratory measurements on non-LoRaWAN LR-FHSS devices have been used to isolate specific operational states, such as radio preparation and transmission, to build analytical models for ToA and current consumption across varying transmit powers and payload sizes [15]. Similarly, the authors in [8] enabled a complete LoRaWAN network (including an ED, GW, and NS) used to carry out real hardware energy consumption measurements of a Class A LoRaWAN ED using LR-FHSS. They utilized this setup to empirically derive power profiles and ToA. Subsequently, the authors used the same testbed to evaluate the PDR and RSSI of LR-FHSS and LoRa in urban and semi-urban scenarios [29]; the urban measurement campaign in [30] reports PRR and RSSI.

Real-world field campaigns have also played a critical role in testing the limits of LR-FHSS. Early validation using commercial EDs and GWs provided foundational insights into the modulation’s practical range and robustness against interference during suburban field measurements with the GW on the roof of Semtech’s Neuchâtel office in Switzerland [25]. In an urban scenario, experiments were conducted in indoor and static conditions, with four EDs and one GW (note: in [32], two of the EDs were located at a greater distance from the GW). More challenging testing scenarios involved sending ED-equipped balloons to the edge of space, successfully transmitting experimental data to two ground-based GWs [24,32]. Satellite-pass measurements in the DEWASAT-1 case study use a weather-station terminal to compare LoRa-CSS and LR-FHSS reception [43].

Finally, several studies bridge the gap between commercial hardware and flexible receiver design by employing software-defined radios (SDRs). By combining commercial EDs with an SDR-based GW utilizing a Hack-RF One frontend, researchers successfully tested novel frequency-hopping techniques in a real indoor network [46]. In [17], an SDR receiver was developed using packet traces captured from a real LR-FHSS transmitter over a short-distance line-of-sight (LoS) link. Similarly, the authors in [44] use SDR for the EDs and the GW. For the latter, the authors develop a cross-technology communication (CTC) solution that enables emulating the LoRa downlink signals (e.g., ACKs) by using LR-FHSS transmission. They deploy 5 EDs and a GW in a non-line-of-sight (NLoS) suburban metropolitan scenario.

## 4. State of the Art: LR-FHSS Performance

This section provides an overview of the main findings in the literature regarding LR-FHSS performance. The section is organized into subsections based on crucial performance parameters, namely delivery, loss, and error rates; energy consumption; network capacity; throughput/goodput; and ToA/latency. Interference recognition is evaluated using exact accuracy (correct identification of the complete interferer set), label accuracy, and category-wise recall [47].

### 4.1. Delivery, Loss, and Error Rates

Most of the research literature on LR-FHSS evaluates reliability using either success measures, such as PDR, PRR, probability of successful transmission, and frame or message delivery probability, or failure measures, such as PLR, PER, and outage probability. Table 7 indicates the metric reported by each study. The documents that belong to this group are [7,9,10,11,12,17,18,22,24,26,37,40,41,42,43,46].

This subsection is organized as follows. First, we define the metrics used in the considered works. Secondly, we review works that evaluate the performance of LR-FHSS. Finally, we focus on works that aim to improve the performance of LR-FHSS.

#### 4.1.1. Metric Definitions

PDR and PRR quantify successfully delivered packets relative to transmitted packets under each study’s counting and reception criteria. A measured ratio estimates success probability under the evaluated conditions, whereas packet delivery success probability denotes the modeled probability of a specified event. Message delivery probability and message success probability refer to the probability of successful delivery of application messages rather than individual frames [22].

PER, or packet error probability in analytical work, is the ratio of packets failing reception or decoding criteria over all sent packets; outage probability corresponds to the probability of a failure event specified by a link model or a decoding model. Note that payload errors or header miss-detection lead to packet reception failure [35]. BER is a bit error metric, which can be used to determine PER. Higher success measures and lower failure measures indicate better reliability.

Packet loss rate (PLR) is the fraction of transmitted packets not successfully received [45,52]. For identical packet populations and reception criteria, PLR=1−PDR. PLR equals PER only when both account for the same type of failures; lower PLR indicates better reliability.

On the other hand, $1-P_{\mathrm{out}}$ denotes non-outage probability and represents packet success probability only when outage is defined as failure of the same corresponding packet event. Comparisons among different research works are expressed in terms of the same metrics used therein.

#### 4.1.2. LR-FHSS Evaluation

Analysis and simulation in [7] yield a packet delivery success probability of approximately 70% for 50,000 EDs using DR8 and sending four packets per hour to a single GW. DR8 outperforms DR9 below approximately 150,000 EDs; above this threshold, DR9 has the higher success probability, although it remains below 0.1. At high offered loads, the collisions caused by DR8’s greater redundancy outweigh its decoding benefit. A similar load-dependent trade-off is reported in [9,22].

Assuming the same scenario and traffic offered per ED as in [7], the authors in [22] employ analysis and simulation in order to evaluate how using frame-level or fragment-level replication mechanisms increases message delivery probability compared with the default operation. They show how frame-level replication outperforms fragment-level replication, with the transmission of three message replicas yielding a message delivery probability very close to 1 for 50,000 EDs using DR8. Also assuming the scenario and conditions from [7] as a baseline, the authors in [9] use analysis and simulation to determine that ACRDA improves average network success by up to 60% compared with default LR-FHSS, reaching values close to 1 for DR8 and 50,000 EDs and approximately doubling the number of EDs supported at an average network success of 0.7.

Advancing these theoretical models further, several studies incorporate real-world channel impairments such as noise and fading. Under these conditions, a single satellite GW can meet a target outage probability of $10^{-2}$ for 60,000 to 120,000 EDs sending 48-byte packets hourly using US DR6 and DR5, respectively. Furthermore, US DR5 enables an extended communication range of up to 1200 km, surpassing US DR6 by roughly 250 km [10]. In a related satellite-based IoMT context, simulations show packet transmission success probability beyond 0.9 and 0.7 for 50,000 and 100,000 EDs sending data to a GW, for a range of distances between ∼800 km and ∼2200 km, considering the presence of noise, and a 2-byte payload size (which corresponds to a medical application) [26]. Unfortunately, the work does not indicate the sending message rate of an ED. To ensure the mathematical accuracy of such extreme-scale simulations, robust analytical models are paramount. Demonstrating this, ref. [37] validated the MC^2^AMB model over a 35-channel grid, proving it highly accurate and superior to the traditional BBM. In high-density tests involving up to 60,000 nodes sending 10-byte packets at 0.002 packets/s, the model correctly predicted that DR8 excels in moderate traffic, while DR9 is better for heavy congestion. In low-density tests (1 packet every 3 h) and constrained networks (reduced to 3 channels, 1 packet/400 s), the BBM critically failed, whereas MC^2^AMB remained precise. Furthermore, these configuration tests verified Semtech’s industry standard, proving that transmitting exactly three header replicas maximizes packet success probability in the evaluated configurations. For the evaluated LEO links, LR-FHSS and LoRa SF12 share the same modeled outage, while HAPS permit lower LoRa SFs [41].

Complementing theoretical simulations, extensive real-world measurement campaigns have verified the practical superiority of LR-FHSS over traditional LoRa in various environments. High-altitude balloon experiments transmitting to ground GWs up to 30 km away achieved an average PDR of roughly 70%, demonstrating that increasing transmit power from 2 dBm to 16 dBm significantly bolsters reliability from 57% to 75% [24,32]. In urban scenarios with ED-GW distances of up to 600 m, these studies confirmed that LR-FHSS consistently outperforms standard LoRa DRs (DR0 to DR6), although it remains highly sensitive to the chosen CR [32]. Further urban and semi-urban evaluations across multiple distances corroborate this performance gap. The authors in [29] provide PDR results obtained experimentally in urban and semi-urban scenarios comprising EDs located at 15 different distances from a GW. They evaluated all LR-FHSS DRs, along with LoRa DR0 and DR5. In the considered scenarios, LoRa DRs lost connectivity beyond 1.5–2 km, whereas LR-FHSS offered high PDR (even exceeding 90%) at 3–4 km. DR10 and DR8 achieve the highest PDR, due to their greater PHY redundancy. While DR11 and DR9 offer moderate PDR in challenging scenarios, they still outperform the PDR of LoRa DR0 while also exhibiting lower ToA and energy consumption. This resilience is echoed in North American urban testbeds, specifically in an experimental measurement campaign in urban scenarios in Halifax (Canada) [30]. The authors investigate the performance of US DR5 and US DR6 LR-FHSS DRs, along with that of US LoRa SF10. To this end, they set up a complete, real-world LoRaWAN network supporting LR-FHSS. In their scenarios, US DR5 outperforms US DR6 and US LoRa SF10 in terms of PRR. At 1.5 km, the reported PRRs are 85% for US DR5 and 75% for both US DR6 and US LoRa DR0 (SF10), with 22 dBm transmit power and a 4-byte payload. Field measurements are also one of the main focuses of the authors in [25], where the FCC configuration corresponding to US DR5 (CR 1/3 and three headers) yields 4% PER at 6.3 km in the measured suburban link, with 14 dBm transmit power. The DEWASAT-1 measurements show higher LR-FHSS packet counts in two of three passes, while LoRa-CSS performs better in the first [43].

#### 4.1.3. LR-FHSS Evaluation and Improvement

To improve the fundamental decoding capabilities of LR-FHSS, several studies have focused on advanced transceiver designs and robust detection schemes. A foundational transceiver design evaluated under AWGN achieved a PER below $10^{-3}$ when the ratio of energy per transmitted symbol to noise density ($E_{s}/N_{0}$) exceeded 6 dB [14]. This architecture was later refined to mitigate co-channel interference (CCI) and Doppler effects; empirical tests involving eight transmitters and one receiver yielded a PER below $10^{-2}$ for DR9 and approaching $10^{-4}$ for DR8 [18]. Addressing severe mobility, authors in [35] evaluated a novel header detector, measuring miss detection probability against $E_{b}/N_{0}$ and demonstrating robust performance even under extreme Doppler rates of up to 250 Hz/s. Other efforts have prioritized receiver simplicity. A low-complexity symbol-by-symbol detector incurs BER performance penalties of 1 dB in AWGN and 1.5 dB in shadowed-Rice fading relative to the optimal receiver [19]. Further validating custom receiver designs, real-world experiments with four EDs at sub-kilometer distances achieved a near-100% PRR [17]. Subsequent trace-driven simulations across diverse environments, including AWGN, the ETU model of terrestrial multipath propagation, and NTN, revealed that maintaining a ∼95% PRR for all channel types requires a minimum SNR of −20 dB for DR8 and −16 dB for DR9.

Beyond core receiver architectures, researchers have proposed targeted PHY techniques to mitigate errors and manage interference. Recognizing that header errors are the primary cause of packet loss, the implementation of network-coded header replication was shown to decrease the PER by more than an order of magnitude for DR8, alongside significant improvements for DR9 [11]. Similarly, applying interference blanking techniques at the receiver can enhance decoding performance and reduce the PER by over an order of magnitude [12]. Frequency and channel allocation strategies also play a critical role. DFH increases PRR by up to a factor of 2.76 in the evaluated real-world environments [46]. In a complementary approach, optimally allocating orthogonal physical channels for headers and payload fragments consistently improved transmission success probabilities across all tested scenarios compared to standard LR-FHSS [28].

Macroscopic and network-level strategies have been evaluated to sustain reliability in highly congested deployments. Fragment-level macro-diversity has proven highly effective, significantly outperforming traditional single-GW reception. This strategy improves peak goodput by up to a factor of five and sustains an 80% packet success probability at higher offered loads than the nearest-GW baseline [34]. At the device transmission level, concatenation techniques offer substantial benefits in dense environments, achieving up to 44% higher message success probability at 50,000 EDs. Conversely, strategies such as embedded retransmission are better suited to low-to-moderate-density networks, as their relative advantages diminish rapidly as device counts scale [40]. Visibility- and load-aware joint LoRa/LR-FHSS access also improves PDR under favorable visibility with additional orthogonal LoRa resources [42].

The authors in [44] provide LR-FHSS PRR results in an experimental testbed deployed in a suburban metropolitan area with NLoS. In such a scenario, LR-FHSS yielded a PRR of 79% at a distance of 2 km between EDs and a gateway. The authors achieve a 15% PRR improvement for uplink-only LR-FHSS devices by repurposing LR-FHSS signals sent by the GW as LoRa ACKs, thus enabling frame retransmission for uplink-only devices.

### 4.2. Energy Consumption

Several studies evaluate the energy performance of LR-FHSS using metrics such as current and power consumption [8,15,25], energy efficiency and energy cost [8,22,23,40], and overall energy consumption [27,42], as can be seen in Table 8.

A critical aspect of LR-FHSS deployment is energy consumption and battery lifetime, which several studies have modeled using real hardware measurements. By profiling devices connected to fully operational networks, researchers have derived precise current consumption models. For example, assuming a 230 mAh battery and a transmission interval of 50 min, the estimated battery lifetime is 2.5 years for DR9/DR11, dropping to 1.2 years for the more robust DR8/DR10 [8]. By evaluating the impact of payload size and ACK modes on Class A LoRaWAN devices, researchers have quantified these efficiency gains; specifically, utilizing unconfirmed (unacknowledged) transmissions lowers the overall current consumption by approximately 3% compared to standard acknowledged communications. Scaling up the hardware profile to a 2400 mAh battery and a 14 dBm transmit power, the analytical model predicts a lifetime exceeding 4.2 years for 10-byte LR-FHSS transmissions every 30 min [15].

When benchmarking against traditional LoRa, the results highlight the energy efficiency of LR-FHSS. Energy profiling reveals that LR-FHSS highly redundant DRs come at a direct cost to efficiency. They generally consume power similarly to the most robust LoRa setting (DR0), making them less energy efficient than faster LoRa alternatives like DR5 [8]. However, because of lower redundancy and shorter ToA, the energy cost per transmitted bit for DR9/DR11 was found to be up to 2.5 times lower than that of DR8/DR10. Deeper analysis incorporating SNR and collision impacts reveals that LR-FHSS DR9 is, overall, more energy efficient than standard LoRa DRs in moderately active networks, with DR0 offering the worst energy efficiency, for a notification period of up to 30 min [15]. Industry models corroborate this: the work in [25] uses a simple model to determine that the annual electric charge consumption of an LR-FHSS ED is 323 mAh and 258 mAh for CR 1/3 and CR 2/3, respectively, considering 6 reports per day with a 20-byte PHY payload. Similar calculations are also provided for LoRa SF10 and SF12, leading to 205 mAh and 280 mAh, respectively, assuming no packet repetitions beyond the original one. The energy performance found for SF12 (i.e., DR0), better than that of CR 1/3 and worse than that of CR 2/3, is consistent with the results found in other works [8].

Beyond hardware baselines, researchers have explored how specific protocol modifications and resource allocation strategies impact energy efficiency, highlighting an inherent trade-off between transmission robustness and energy savings. For instance, while message replication drastically improves reliability, transmitting without replication is nearly four times more energy efficient than sending three replicas [22]. Conversely, reducing protocol overhead directly extends battery life, as demonstrated by the implementation of payload concatenation techniques [40]. To actively balance these parameters, advanced probabilistic strategies dynamically enhance reliability and performance. Before each transmission, a device randomly selects a configuration setup (comprising the number of header replicas and CR) based on a setup probability distribution provided by the NS [23]. For the evaluated 10-byte payloads and networks with up to approximately 140,000 EDs, the energy-efficiency optimum favors configurations S1 and S2, which use fewer header replicas and payload fragments. Crucially, maximizing overall network goodput does not guarantee optimal energy efficiency; operators must actively balance these two metrics based on their deployment priorities, allowing this dynamic strategy to significantly outperform fixed, static configurations. Joint access can also reduce the modeled transmission energy by using shorter LoRa transmissions when feasible [42].

Finally, macro-level network architecture plays a vital role in overall device energy consumption. Going beyond the default single-tier star topology, analysis and simulation of two-tier architectures demonstrate notable energy savings [27]. By allowing devices to transmit over shorter distances to intermediate nodes, two-tier networks inherently save energy. In these multi-tier comparisons, LR-FHSS continues to prove more energy efficient than standard LoRa by supporting a substantially larger volume of devices while simultaneously reducing the frequency of energy-wasting packet collisions.

### 4.3. Network Capacity

Network capacity is evaluated in several studies, typically in terms of the number of EDs that can be supported within a given area or under specific traffic conditions. When evaluating absolute network capacity, LR-FHSS demonstrates a massive improvement over traditional LoRa. Table 9 offers a summary of these documents, which consistently show that LR-FHSS can support significantly more devices than traditional LoRa, making it suitable for dense IoT deployments.

To establish the fundamental scalability limits of LR-FHSS, researchers have developed detailed analytical models that define baseline network capacities. By distinguishing aligned subchannels (i.e., subchannels without interference) from overlapping subchannels, the model estimates that an LR-FHSS GW can serve over 18,000 EDs with aligned subchannels, using DR8 and 10-byte payloads at maximum-capacity traffic [31], surpassing earlier estimates of approximately 16,000 EDs [6]. This work also optimized protocol parameters based on payload size and CR, finding that while the default two header replicas are optimal for DR9 and DR11 (for payloads under 58 bytes with a CR 2/3), maximizing payload channel utilization for DR8 and DR10 requires increasing the replicas to four, five, or even six, depending on the payload size. For instance, using a CR 1/3, four header replicas improve utilization for payloads of 22 bytes or larger, with four header replicas improving utilization for 72% of packets in the considered CampusIoT dataset [31]. In the context of DtS communications, detailed outage probability analyses indicate that LR-FHSS can support up to 60,000 or 120,000 EDs per hour sending 48-byte packets, depending on the chosen DR, at a target outage probability of $10^{-2}$ [10].

Building upon these baselines, numerous studies have proposed PHY and resource allocation enhancements to increase supported device density. Specialized receiver designs employing interference blanking techniques have demonstrated the ability to increase decoding capacity by roughly 2.5× for 250 concurrent signals [12]. This work emphasizes that improvements in spectral efficiency and interference management are fundamental to increasing the density of supported devices. Similarly, implementing orthogonal physical channel allocation schemes can boost client capacity by up to 50% while maintaining the same reliability level [28]. Further optimizations focus on hopping behavior and demodulator allocation; combining novel Wide-Gap (li-fan) FHSs with strategies like “Early-Decode” and “Early-Drop” has yielded up to a 50% increase in decoding capacity. In particularly high-traffic scenarios, these li-fan sequences alone outperform conventional driver sequences by as much as 100% [38].

At higher protocol layers, advanced access schemes and payload strategies further alleviate channel congestion. The application of the ACRDA protocol and its verification against collision by implementing GW-level interference cancellation, resolves collisions that would otherwise cause data loss. This mechanism allows the system to support a massive number of concurrent transmissions, scaling up to tens of thousands of devices per GW or more than two-fold compared to the unmodified technology [9]. This theoretical massive scalability has been corroborated by real-world packet traces, confirming that the protocol meets its high-capacity expectations in practice and suggesting customized receiver methods that can further enhance performance beyond standard reported values [17]. To effectively manage congestion at scale, packet-level aggregation is also highly beneficial; employing payload concatenation reduces the total number of competing transmissions, whereas replication or embedded retransmission strategies exacerbate channel congestion and lengthen the per-packet ToA, limiting their effectiveness in high-capacity environments [40].

Finally, capacity enhancements are not limited to the PHY or receiver algorithms but can also be achieved through architectural innovations. As demonstrated in [13], integrating D2D communications can significantly reduce the collision probability on the direct satellite uplink. By offloading traffic through relays and utilizing different frequency resources, this hybrid approach effectively increases the overall available spectrum and system robustness. Consequently, this mechanism reduces the contention load on the satellite GW, allowing a larger aggregate number of devices to share the network resources efficiently compared to a purely direct access topology. Specifically, compared to standard LR-FHSS, the D2D-aided variant demonstrates, for a target outage probability of $10^{-2}$, capacity increases by 249.9% and 150.1% for US DR6 and US DR5, respectively.

### 4.4. Throughput/Goodput

Throughput and goodput are commonly used to evaluate the efficiency with which LR-FHSS transmits usable data over time, as summarized in Table 10. Foundational studies such as [6,25] establish the baseline performance metrics, assessing the raw throughput capabilities of the protocol under standard configurations. These works highlight the trade-offs between on-air time and network capacity, providing a benchmark for the number of packets the system can handle before saturation. Compared with earlier simulations [6], the analytical model in [31] predicts a threefold improvement in goodput under its modeling assumptions.

Building on the foundational works, ref. [12] investigates throughput from the PHY perspective, proposing an advanced receiver design that improves spectral efficiency. By enhancing the detection of FHSs, this approach allows for higher aggregate throughput in massive IoT scenarios, effectively increasing the efficiency of the constrained satellite spectrum.

Beyond raw capacity, significant research focuses on improving goodput, which is the measure of useful data delivered at the application layer without errors, by mitigating packet loss in dense environments. In [9], the authors introduce the ACRDA protocol. This study demonstrates that by employing interference cancellation techniques at the GW, the network can recover colliding packets that would otherwise be discarded. This increase in the successful packet rate directly boosts the effective goodput, particularly in saturated networks where collision rates are high. The authors in [48] also evaluate the use of ACRDA, including a probabilistic header repetition technique, finding that goodput can be maximized with 3 and 4 repetitions. Similarly, the macro-diversity technique proposed in [34] improves packet success probability by combining fragments received at different GWs, thereby improving goodput, with peak goodput up to five times that of the nearest-GW reception baseline. Then, simulation tools, such as the discrete-event simulator presented in [16], are frequently employed to validate these throughput gains, allowing researchers to model complex traffic patterns and evaluate throughput and average network success under the simulated traffic conditions.

The authors in [39] show that their robust SIC method doubles the performance of existing SIC methods. Specifically, testing within a 137 kHz channel showed that the new method achieves capacities of approximately 7.9 kbps for DR8 and 7.4 kbps for DR9 at 90% PRR. Furthermore, these achieved capacities reach within 67% and 62% of the theoretical analytical bound for DR8 and DR9, respectively, indicating that the new receiver design approaches the fundamental performance limits of LR-FHSS networks.

Finally, optimization strategies play a critical role in maximizing goodput by balancing reliability against redundancy. To maximize network goodput, researchers have extensively investigated the optimal configuration of header replication and CR. The authors in [23] propose a probabilistic resource allocation method, dynamically adjusting the payload CR and the number of header copies. By mathematically modeling how different transmission setups influence the generation rate of headers and payload fragments, as well as the resulting collision risks during vulnerable periods, the NS can fine-tune setup probability distributions to target specific operational goals. This approach ensures that the network does not waste airtime on unnecessary redundancy while maintaining high reliability, thereby maximizing the ratio of useful information transmitted. Simulations of this probabilistic allocation highlight a fundamental trade-off: maximizing overall network goodput necessitates a higher number of header replicas and a diverse mix of configuration setups across devices. For joint LoRa/LR-FHSS access, stricter access thresholds improve PDR but reduce unique payload receptions across a satellite pass [42].

Similarly, header redundancy optimization balances header overhead against decoding success [31]. The dependence on payload size and CR is detailed in Section 4.3, with the evaluation method and a representative outcome summarized in Table 12. In a broader architectural context, ref. [27] analyzes these efficiency metrics within a two-tier network architecture, evaluating how relaying data affects the overall system throughput compared to direct access.

### 4.5. ToA/Latency

ToA and latency are critical metrics for evaluating the responsiveness of the LR-FHSS protocol, particularly given its design for long-range, DtS links. These metrics are rigorously examined in [8,15,25,26,40,43], for which a comparative summary is provided in Table 11. A foundational analysis is established in [8,15], where the authors derive experiment-based analytical models to quantify airtime. Their findings demonstrate that LR-FHSS generally results in a longer ToA compared to legacy LoRa. This increase is primarily attributed to the protocol’s PHY mechanisms, specifically, the redundant header transmission, which reduces the effective data rate to ensure robustness against interference and channel fading. Because of lower redundancy and shorter ToA, the energy cost and channel occupation for faster rates like DR9/DR11 were found to be significantly lower than those of the robust DR8/DR10. For a 20-byte payload with cyclic redundancy check (CRC), the DEWASAT-1 study lists LR-FHSS ToAs of 1.868 s and 1.051 s for CRs 1/3 and 2/3, respectively [43].

The practical implications of these latency characteristics are further detailed in [25]. This technical analysis indicates that while on average LoRa provides lower latency, the higher reliability of LR-FHSS provides a better worst-case scenario, and thus a more stable metric. In the specific application for IoMT illustrated in [26], the authors highlight that while LR-FHSS is viable for periodic reporting of health monitoring systems in remote areas, the increased latency, compared to LoRa, can hinder frequent updates. They also propose a method where the header transmission is omitted to reduce ToA.

Finally, latency is one of the metrics that the authors in [40] use to test their three proposed mechanisms: concatenation, embedded retransmission, and replication. The maximum delays for the three methods and the average delay of the concatenation technique remain stable as the network scales. Concatenation incurs the highest delays as a trade-off for high reliability. Replication offers the lowest average delay, while embedded retransmission has a higher average delay. However, when configured with matching parameters, both replication and embedded retransmission share the same maximum delay, making them significantly faster than concatenation.

## 5. State of the Art: LR-FHSS Communication Improvement Techniques

Beyond performance evaluation, some studies on LR-FHSS explore how to improve LR-FHSS communication by modifying the operation of either the receiver or both the transmitter and the receiver.

Table 12 links the LR-FHSS improvement techniques discussed throughout this survey to their evaluation methods (Section 3), reported outcomes (Section 4.1, Section 4.2, Section 4.3, Section 4.4 and Section 4.5), and application contexts (Section 6). Related receiver variants are grouped, and resource-allocation, relaying, and coexistence techniques discussed in other sections are also included. The table complements the complete study inventory in Table 3; the following subsections explain how the main LR-FHSS improvement techniques operate.

### 5.1. Modified Receiver Techniques

Many literature contributions go beyond LR-FHSS settings and propose updated PHY techniques intended to improve LR-FHSS communication performance (Table 12). Most such techniques focus on enhancing only receiver-side (i.e., GW-side) operation. These include headerless frame recovery [20,21]; low-complexity detection [19]; interference recognition [47]; and decoding signals in the presence of interference [9,12,17,39] or interference and Doppler effects [18].

Advancements in LR-FHSS receiver design have largely focused on addressing the challenges of header loss and severe Doppler shifts, which are particularly critical in LEO satellite scenarios. The authors in [21] and in a subsequent work [20] developed improved LR-FHSS receiver techniques specifically designed to recover frames without headers (i.e., frames whose headers have been lost, which primarily happens due to collision in congested scenarios). The initial work in [21] was based on the Greedy Headerless Decoding heuristic. This technique scans through the received time-frequency fragment matrix, trying to match fragment sequences to known FHSs. If a match is found, the fragment sequence is added to the list of recovered frames. Another approach pursuing the same goal, based on an ILP model, was also considered, but was found to be more complex and time-consuming [21]. However, the Greedy Headerless Decoding heuristic did not consider Doppler effects, which produce frequency shifts in LEO satellite scenarios. For this reason, authors extended the work by adding a mechanism to compensate for such effects [20]. Table 12 summarizes the evaluation approach and the distinction between the original and Doppler-aware variants. Further addressing the Doppler challenge, Vangelista et al. [35] proposed a highly resource-efficient, two-stage header detection scheme—comprising an energy peak finder and a precise header locator. Its reported complexity reduction and evaluated Doppler range are summarized in Table 12.

Maleki et al. [19] exploit the predictable phase behavior of GMSK to detect symbols individually instead of processing the sequence with the Viterbi algorithm. This reduces computational complexity at the cost of the BER penalties reported in Section 4.1 and Table 12. The authors in [14] introduced the first dedicated LR-FHSS receiver capable of simultaneously processing up to 3120 frequency-hopping channels. They later refined this design [18] to better mitigate CCI and Doppler effects. Their performance evaluations confirmed that the GMSK-based LR-FHSS system achieves superior synchronization accuracy, lower miss-detection probability, and reduced PER compared to a baseline quadrature phase-shift keying architecture.

In dense IoT deployments, mitigating interference and resolving collisions remain paramount. One effective strategy is interference blanking, introduced in [12], which is based on setting the values of signal portions damaged (i.e., by interference) to zero, essentially ignoring or “blanking” them out. In consequence, the decoder avoids using unreliable information during the decoding process, which contributes to recovering the original bits more accurately. Different approaches were evaluated, with using weighted signal values based on estimated signal-to-interference-plus-noise ratio providing the best performance. Moving toward active interference cancellation, J. Bukhari et al. in [17] developed an SDR receiver design based on data that they captured while performing real LR-FHSS transmission experiments. The SDR-based LR-FHSS receiver includes a component that is in charge of signal reconstruction, which performs waveform regeneration and SIC to enhance packet detection and decoding. SIC is based on subtracting the waveform of a decoded packet to eliminate the interference it produces to other packets. Additionally, a new method, IPAD, was proposed for improved error correction decoding. IPAD relies on a priori knowledge of collisions, which is possible for all packets with correctly decoded headers, leading to knowing the entire time–frequency occupancy map. If a symbol is subject to high interference, it should be given less confidence to minimize misleading the decoder. The associated PRR gain and evaluation method are summarized in Table 12. The same authors in [39] introduce an updated and more robust SIC technique, which effectively estimates channel gain in dense collision scenarios. It achieves this through multi-symbol averaging, a process that inherently suppresses random interference while allowing the desired signal components to accumulate constructively. Xu et al. [47] fuse local time–frequency and frequency–domain features to recognize coexisting interferers, including combinations absent from training.

Furthermore, the application of a modified ACRDA protocol (from [53]) to LR-FHSS has shown significant promise [9]. In ACRDA, the sender transmits several replicas of a message. Once the receiver successfully decodes one replica, it can apply interference cancellation in order to suppress the effect of the remaining replicas. Therefore, the collisions that would have been produced by such replicas will be avoided. Table 12 summarizes the reported reliability and throughput gains. The study also reports reduced ED energy consumption and support for more devices at a target reliability. However, as a trade-off, the GW requires more memory and processing power compared to the standard LR-FHSS.

Under the assumption of uplink-only LR-FHSS devices, the authors in [44] develop a CTC solution that repurposes LR-FHSS signals sent by the GW as LoRa ACKs, thus extending the link range for downlink transmissions and enabling frame retransmission for such devices. For confirmed traffic, a PRR increase of 15% is achieved.

Beyond individual receiver enhancements, the recent literature explores system-level architectures and network coexistence. Introducing structural changes at the network architecture level, the work in [34] proposes a fragment-level macro-diversity strategy where multiple GWs collaboratively receive LR-FHSS fragments. In this scheme, once a GW decodes a header, it alerts a centralized controller, which derives the hopping sequence and orchestrates all GWs to track the associated payload fragments. By aggregating these fragments at the controller for de-duplication and reconstruction, the proposed strategy achieves substantial capacity and goodput improvements over conventional single-GW baselines.

### 5.2. Modified Transmitter and Receiver Techniques

A few proposed LR-FHSS improvement techniques involve both the transmitter and the receiver (i.e., the ED and the GW), namely FHS management [38,46], message or fragment replication [22], and optimal physical channel allocation [28].

Based on real-world measurements, the authors in [46] found that dynamic interference is one of the main reasons for diminished transmission performance. They determined that the choice of FHS directly affects error patterns, and if it leads to bursty errors, forward error correction may fail to recover the data, resulting in packet loss. For this reason, the authors introduced DFH, which adapts frequency hopping to real-time channel conditions. DFH, which runs at the GW, predicts the PRR of each FHS based on per-hop SNR, using a model calibrated separately for each ED. Then, an interleaving-based search algorithm identifies candidate FHSs, while conditional blocking of particularly poor sub-channels and greedy FHS selection across nodes further improve reliability. The measured PRR gain is summarized in Table 12. However, DFH introduces computational overhead at the GW and requires downlink signaling; the authors assess these costs and propose on-demand FHS updates to limit signaling overhead.

Given the importance of reliability in LR-FHSS, the authors in [22] propose two improvement approaches based on message replication, known as frame replication and fragment replication. Frame replication sends the entire message multiple times as separate, identical frames. The receiver only needs to correctly receive one copy. On the other hand, fragment replication sends the message in a single frame, but each payload fragment is repeated multiple times within that frame. The receiver combines the repeated fragments to improve decoding reliability. To model the two proposed methods, the approach used in [7] is adopted, allowing for the analysis of both message delivery probability and energy efficiency. After performing mathematical analysis, simulations were carried out to verify the results. Two DRs, DR8 and DR9, were considered in the simulations. The results show that as the number of nodes in the network increases, the performance of all schemes declines due to the higher interference caused by the increased traffic. However, both frame and fragment replication schemes outperform systems without replication, with more replications leading to higher message delivery probability. In terms of energy efficiency, DR9 in the no-replication scenario offers the best performance in sparse traffic, while the same DR but with fragment replication is more efficient in moderate-to-heavy traffic. It was also observed that transmitting replicas as separate frames provides better reliability in low-traffic scenarios, while fragment replication is more effective in high-traffic scenarios. This happens because, under high traffic, sending full message replicas can lead to congestion. Another approach to increase the performance of LR-FHSS networks relies on modifying frame header placement, since interference leading to reception failure of the repeated headers is typically correlated [45]. Finally, the authors in [48] propose using probabilistic header repetition, increasing the average number of header repetitions, yielding a goodput increase. This study also assumes the use of SIC.

Reliability and density of users can also be increased via the optimal allocation of physical channels for each type of fragment (header or payload). The authors in [28] propose a set of optimization strategies to optimally distribute physical channels between headers and payload fragments. They take into account at least three parameters: the payload size, the DR, and the number of devices in the network.

Sophisticated packet-level transmission policies can be dynamically applied to balance reliability, latency, and channel congestion. Recent research introduces three distinct strategies tailored to varying network conditions: concatenation, embedded retransmission, and replication [40]. For delay-tolerant applications, concatenation aggregates multiple messages into a single transmission, effectively minimizing protocol overhead and alleviating network congestion at the cost of higher latency. When a balance between speed and reliability is required in less crowded networks, embedded retransmission offers a practical middle ground by piggybacking previous messages onto new packets. Conversely, pure replication enhances baseline reliability by transmitting several independent duplicates of each packet; however, this approach risks overloading the channel and exacerbating collisions in high-density deployments. At the MAC layer, joint-access policies select LoRa or LR-FHSS using satellite visibility and estimated contention [42].

In [52], the authors propose segment-level coded frequency hopping (SLC-FH) over high-order Galois fields, with parity-based erasure detection to combat clustered errors. The authors report support for approximately 10× more users at a packet loss rate of $10^{-3}$ and roughly 50% lower energy consumption than a BCH-coded baseline at comparable reliability. However, the assumptions underlying the analytical models limit direct comparison with standard LR-FHSS.

Finally, a variety of techniques are proposed in [38]. For the transmitter side, they propose a novel FHS design that leverages Wide-Gap sequences to optimize collision resilience and align perfectly with LR-FHSS regulatory constraints. On the receiver side, the authors introduce two demodulator allocation strategies, called “Early-Decode” and “Early-Drop”, to maximize the efficiency of decoding resources. The “Early-Decode” mechanism optimizes processing by freeing up a demodulator as soon as it has successfully gathered enough convolutional blocks to decode a frame. Conversely, the “Early-Drop” strategy proactively aborts the reception of a packet and liberates the processor if a specific threshold of fragment losses is reached, effectively preventing wasted computational effort on unrecoverable frames. Furthermore, they also introduce a “header tolerance” method, which allows for minor collision overlaps to be processed, yielding immediate improvements in payload recovery.

## 6. State of the Art: LR-FHSS-Enabled Applications

This section identifies the main LR-FHSS applications envisioned in the literature. The section first focuses on DtS-IoT applications as the main motivating area for LR-FHSS researchers. Secondly, works on terrestrial LR-FHSS applications are highlighted. Finally, other general or more specific LR-FHSS use cases are provided. Table 12 connects these application contexts to the LR-FHSS improvement techniques reviewed in the previous section and their evaluation evidence.

### 6.1. DtS-IoT

Many of the reviewed studies focus on DtS-IoT, presenting LR-FHSS as a suitable technology for connecting devices in remote areas where ground infrastructure is limited or unavailable. The works in [7,8,9,12,13,15,17,18,19,20,21,22,24,26,28,31,32,35,37,38,39,40,42,43] point to the ability of LR-FHSS to deliver reliable long-range communication, even in challenging conditions like low SNR, interference, and severe Doppler rates, which are common in satellite links. Interference recognition also targets DtS-IoT receiver monitoring [47].

### 6.2. Terrestrial Networks

Though not as numerous, some studies apply LR-FHSS in real-world terrestrial networks, evaluating its performance in rural, urban, mobile environments, and even in indoor scenarios. These works often compare LR-FHSS with LoRa, demonstrating its improved range and delivery success in environments vulnerable to interference or mobile conditions. Real-world scenarios, such as those in [11,24,29,30,32], validate LR-FHSS performance under realistic terrestrial conditions, suggesting its potential for reliable long-range coverage even without satellite infrastructure.

### 6.3. Others

Several papers investigate both general and specialized IoT domains beyond satellite and terrestrial communication. The studies in [16,23,25,27,48] include broad sensor networks and large-scale monitoring systems, whereas the one in [26] focuses on IoMT. In these cases, LR-FHSS is valued for its scalability, energy efficiency, and ability to maintain communication in sparse or constrained environments. These studies demonstrate that LR-FHSS is being considered for a diverse range of IoT applications requiring robust, low-power, long-range communication. HAPS-based remote-area IoT coverage is also considered in [41].

## 7. Challenges and Future Directions

While LR-FHSS significantly extends the capabilities of traditional LoRaWAN deployments, several technical hurdles remain. This section presents current challenges and proposes several future research directions in the field of LR-FHSS.

### 7.1. Current Challenges

#### 7.1.1. Protocol Limitations in Initial Access and Downlink Transmissions

A fundamental operational bottleneck in the current LoRaWAN protocol is the requirement to perform the initial *Join* procedure and configuration phase using standard LoRa modulation, rather than with the more robust LR-FHSS. In long-distance or satellite applications, this discrepancy leads to suboptimal performance, as devices may struggle to successfully join the network even if they are capable of successfully delivering LR-FHSS payloads once connected. Moreover, using LoRa degrades the number of connections that a GW can handle, especially considering that it is also used for any downlink transmission. While creative solutions to this problem have been recently proposed [44], a stable solution should be included in future LR-FHSS standardized developments.

#### 7.1.2. Interference in Overlapping Networks

As IoT deployments continue to scale, the spatial and spectral overlap between DtS LR-FHSS networks and existing infrastructure will increase. This coexistence inevitably creates cross-technology interference that extends well beyond standard LoRa to include LoRaWAN deployments utilizing FSK modulation, as well as any other technology operating within the same spectrum, such as Sigfox. Because these diverse technologies must share the same license-free industrial, scientific, and medical (ISM) bands, avoiding and/or mitigating the impact of collisions is a significant challenge for network reliability. Promising approaches include GW-received signal filtering [33] and the allocation of dedicated channels for LR-FHSS.

#### 7.1.3. Doppler Effects in LEO Satellite Links

In DtS architectures, LEO satellite links experience large, time-varying Doppler shifts due to the high relative velocity between the satellite and ground nodes (of approximately 7.8 km/s). These PHY characteristics severely complicate header reception. While some works have tackled this problem, compensating for these severe Doppler effects requires highly computationally intensive signal processing at the receiver, which currently limits the practical scalability and efficiency of the DtS architecture.

### 7.2. Future Directions

#### 7.2.1. Advanced Receiver Design and Interference Mitigation

To directly address header synchronization bottlenecks in LEO links and enhance LR-FHSS network scalability, future work should investigate designing advanced collision determination methods, adaptive decoding strategies, and collaborative headerless decoding in multiple GWs.

#### 7.2.2. Exploiting Network Redundancy and Satellite Constellation Design

A promising avenue for mitigating both interference and packet loss is to leverage the inherent redundancy of LR-FHSS reception through multiple GWs. Because LR-FHSS fragments packets across multiple frequencies and time slots, future network architectures could employ advanced macro-diversity and cooperative reception techniques across overlapping terrestrial GWs. Furthermore, future work should also focus on exploring satellite constellation design and multi-satellite connectivity, among other techniques, to develop efficient strategies that grant a specific quality of service in satellite communication scenarios.

#### 7.2.3. Comprehensive System Modeling and Energy Analysis

To successfully support massive LPWAN deployments, extensive system modeling is critical to balance coding redundancy, overall network capacity, interference tolerance, and battery lifetime. Furthermore, as hardware evolves, analyzing the current consumption of next-generation LR-FHSS devices will be essential.

#### 7.2.4. Energy Performance Against Popular LPWAN Alternatives

Comparative research evaluating the energy efficiency and battery lifetime of LR-FHSS against alternative LPWAN technologies, such as LoRa, narrowband IoT (NB-IoT), LTE for machines (LTE-M), and reduced capability (RedCap), will be vital for defining optimal use cases for each standard, also considering other metrics.

#### 7.2.5. Adaptive Protocols

Future research could investigate advanced hybrid schemes that dynamically adapt transmission parameters to current network conditions and delay requirements [40].

#### 7.2.6. Application-Aware Transmission

Application-aware transmission strategies could be developed to prioritize the most relevant data. This approach might even allow the suppression of nonessential messages to reduce traffic congestion and improve overall network reliability, while carefully managing the resulting impact on the application layer.

#### 7.2.7. Experimental Validation

While theoretical models are essential and simulation-based work is extremely valuable, experimental evaluation through real-world deployments and hardware-based testing is crucial to confirm the performance of LR-FHSS and the practical feasibility and effectiveness of the proposed improvement techniques in DtS and terrestrial large-scale LR-FHSS networks. The existing body of research based on real-world experiments should be extended to cover a broader range of environments, notably including actual satellite-based LR-FHSS deployments, together with more diverse device locations, traffic patterns, and network scales.

More specifically, there are two main areas of real-world experiments that need to be prioritized: (i) DtS experiments using LEO satellites and (ii) network capacity experiments. Early satellite-pass measurements are available from the DEWASAT-1 case study [43]. However, further LEO experiments should quantify Doppler-related losses and validate dedicated mitigation techniques at scale [14,18,20,35]. On the other hand, LR-FHSS network capacity studies report that a single GW can support up to tens of thousands of EDs (see Section 4.3). However, according to the existing literature, real-world LR-FHSS experiments are based on networks comprising a single-digit number of devices. Increasing the latter would allow to progress towards confirming the promising results predicted by analysis and simulation. Results from both DtS and terrestrial IoT network environments will be useful.

## 8. Conclusions

As LR-FHSS continues to attract significant attention from the research community, its potential to revolutionize DtS and terrestrial IoT environments is undeniable. To our best knowledge, this article has provided the first consolidated view of the LR-FHSS research landscape, covering the existing body of research literature on LR-FHSS as of the 1st of October 2026. Note that publicly available LR-FHSS overviews (e.g., [6]) describe and/or evaluate LR-FHSS but do not carry out an LR-FHSS research literature study. By synthesizing the current state of the art, we have identified the most pressing challenges currently facing the research community and proposed strategic directions for future work. Ultimately, we hope that this review serves as a foundational roadmap to guide and accelerate future innovations in LR-FHSS technologies.

## Figures and Tables

**Table 1:** LoRaWAN DR profiles for the EU 863–870 MHz band [51].

DR	Modulation	SF	CR	Bandwidth	Bit Rate	Header Copies and Hops	Max. Payload
DR0–DR5	LoRa	SF12–SF7	4/5	125 kHz	250–5470 bps	1 copy, no hops	51–242 bytes
DR6	LoRa	SF7	4/5	250 kHz	11,000 bps	1 copy, no hops	242 bytes
DR7	FSK	N/A	N/A	50 kHz	50,000 bps	1 copy, no hops	242 bytes
DR8	LR-FHSS	N/A	1/3	137 kHz	162 bps	3 copies, 35 channels	50 bytes
DR9	LR-FHSS	N/A	2/3	137 kHz	325 bps	2 copies, 35 channels	115 bytes
DR10	LR-FHSS	N/A	1/3	336 kHz	162 bps	3 copies, 86 channels	50 bytes
DR11	LR-FHSS	N/A	2/3	336 kHz	325 bps	2 copies, 86 channels	115 bytes
DR12	LoRa	SF6	4/5	125 kHz	9375 bps	1 copy, no hops	242 bytes
DR13	LoRa	SF5	4/5	125 kHz	15,625 bps	1 copy, no hops	242 bytes

Note: DR8–DR11 are available only for uplink transmissions. DR14 is reserved for future use (RFU), and DR15 is defined for custom extensions. The values correspond to RP002-1.0.5 [51].

**Table 2:** Uplink DR definitions for the US 902–928 MHz band [51].

DR	Modulation	SF	CR	Bandwidth	Bit Rate	Max. Payload
DR0–DR3	LoRa	SF10–SF7	4/5	125 kHz	980–5470 bps	11–242 bytes
DR4	LoRa	SF8	4/5	500 kHz	12,500 bps	242 bytes
DR5	LR-FHSS	N/A	1/3	1.523 MHz	162 bps	50 bytes
DR6	LR-FHSS	N/A	2/3	1.523 MHz	325 bps	125 bytes
DR7	LoRa	SF6	4/5	125 kHz	9375 bps	242 bytes
DR8	LoRa	SF5	4/5	125 kHz	15,625 bps	242 bytes

Note: These LR-FHSS configurations satisfy the FCC 400 ms dwell-time restriction while maintaining long-range links. “Max. payload” denotes the maximum frame payload when repeaters are not used.

**Table 3:** List of surveyed documents.

Ref.	Year	Doc. Type	Layer	Target Scenario	Proposed Improvement Technique	Evaluation Tools	Performance Metrics
[6]	2021	Journal paper	PHY	Large and dense networks	N/A	Simulation	ED and network capacity
[7]	2022	Journal paper	PHY	DtS-IoT	N/A	Math. analysis, simulation	Packet delivery success probability, capture effect probability
[8]	2024	Journal paper	PHY & MAC	DtS-IoT	N/A	Math. analysis, real experiments	Average current consumption, battery lifetime, energy efficiency
[9]	2024	Journal paper	PHY & MAC	DtS-IoT	Asynchronous contention resolution diversity ALOHA (ACRDA)	Math. analysis, simulation	Average network success, goodput, number of supported EDs
[10]	2023	Journal paper	PHY & MAC	DtS-IoT	N/A	Math. analysis, simulation	Outage probability, network capacity
[11]	2024	Journal paper	PHY	Terrestrial IoT	Network-coded LR-FHSS (LR-FHSS-NC)	Math. analysis, simulation	Packet error probability
[12]	2023	Conf. paper	PHY	DtS-IoT	Interference blanking	Math. analysis, simulation	Payload error rate, network capacity
[13]	2024	Journal paper	PHY & MAC	DtS-IoT	Device-to-device (D2D) aided LR-FHSS	Math. analysis, simulation	Network capacity, ED density
[14]	2023	Journal paper	PHY	DtS-IoT	New transceiver, new 2-bit preamble	Math. analysis, simulation	Miss-detection probability, packet error rate (PER)
[15]	2025	Journal paper	PHY	DtS-IoT	N/A	Math. analysis, real experiments	Current consumption, time-on-air (ToA)
[16]	2024	Conf. paper	MAC	non-terrestrial networks (NTN)	LR-FHSS-Sim	Simulation	Average network success, throughput
[17]	2024	Journal paper	PHY	DtS-IoT	Successive interference cancellation (SIC) and interference power-aware decoding (IPAD)	Math. analysis, simulation, real experiments	Packet reception rate (PRR), network capacity, phase error
[18]	2025	Journal paper	PHY	DtS-IoT	Robust synchronization algorithm	Math. analysis, simulation, real experiments	PER
[19]	2024	Conf. paper	PHY	DtS-IoT	Low-complexity signal detection	Math. analysis, simulation	bit error rate (BER)
[20]	2025	Journal paper	PHY	DtS-IoT	Enhanced decoding techniques	Simulation	Transmission outcome, channel occupancy, F1 score
[21]	2023	Conf. paper	PHY	DtS-IoT	Recovering headerless frames	Math. analysis, simulation	Frame detection probability, computation time
[22]	2024	Conf. paper	PHY & MAC	DtS-IoT	Message replication	Math. analysis, simulation	Message delivery probability, energy efficiency
[23]	2025	Journal paper	PHY & MAC	Dense IoT networks	Probabilistic payload and header replica configuration	Math. analysis, simulation	Goodput, energy efficiency
[24]	2024	Conf. paper	PHY	Spatial and urban environment	N/A	Real experiments	received signal strength indicator (RSSI), packet delivery success, packet delivery ratio (PDR)
[25]	2022	Technical report	PHY & MAC	N/A	N/A	Math. analysis, simulation, real experiments	Capacity, latency, power consumption, battery lifetime, frequency drift tolerance
[26]	2024	Conf. paper	PHY	DtS-IoT for Internet of Medical Things (IoMT)	N/A	Simulation	Probability of successful transmission, ToA
[27]	2024	Conf. paper	PHY & MAC	Large scale IoT	Two-tier network architecture	Math. analysis, simulation	Energy consumption, throughput
[28]	2025	Journal paper	PHY	Dense IoT networks	Orthogonal allocation of physical channels for headers and payloads	Math. analysis, simulation	Success probability
[29]	2025	Journal paper	PHY & MAC	Terrestrial	N/A	Real experiments	PDR, RSSI
[30]	2025	Conf. paper	PHY & MAC	Terrestrial	N/A	Real experiments	PRR, RSSI
[31]	2025	Journal paper	PHY	Dense IoT networks	N/A	Math. analysis, simulation	Goodput, network capacity
[32]	2025	Conf. paper	PHY	Spatial and urban environment	N/A	Real experiments	RSSI, PDR
[33]	2025	Journal paper	PHY	DtS and terrestrial IoT	Filtering-based detector for the LoRa chirp	Simulation	BER
[34]	2025	Conf. paper	PHY & MAC	Large-scale IoT	Multi-GW collaborative reception (Macro-diversity)	Math. analysis	Packet success probability, goodput
[35]	2025	Conf. paper	PHY	DtS-IoT	Low-complexity LR-FHSS header detection structure	Simulation	Header detection probability
[36]	2025	Journal paper	PHY & MAC	DtS-IoT	N/A	Simulation	BER, ToA
[37]	2025	Conf. paper	MAC	DtS-IoT	Multi-class, multi-channel ALOHA-based model (MC^2^AMB)	Math. analysis, simulation	PDR
[38]	2025	Journal paper	PHY	DtS-IoT	Novel Wide-Gap frequency-hopping sequences (FHSs) and two innovative demodulator allocation strategies	Simulation	Collision rate, decoded payloads, decoded data
[39]	2025	Conf. paper	PHY	Terrestrial	New SIC method	Math. analysis, simulation	PRR, throughput (network load)
[40]	2025	Journal paper	MAC	DtS-IoT	New message transmission techniques	Math. analysis, simulation	Message success probability, message delay, energy per successfully delivered message
[41]	2026	Preprint	PHY	LEO satellite and high-altitude platform station (HAPS) IoT	N/A	Math. analysis, simulation	Outage probability
[42]	2026	Conf. paper	MAC	DtS-IoT	Joint LoRa/LR-FHSS access and resource allocation	Math. analysis, simulation	PDR, unique payload throughput, relative transmission energy
[43]	2023	Conf. paper	PHY	DtS-IoT	N/A	Math. analysis, simulation, real experiments	Received packet count, link margin, ToA, bit rate
[44]	2026	Conf. paper	PHY & MAC	DtS and terrestrial IoT	Using LR-FHSS to emulate LoRa downlink signals	Math. analysis, experimentation	PRR
[45]	2026	Journal paper	PHY	DtS-IoT	Header repetition placement modification	Math. analysis, simulation	Packet Loss Rate (PLR)
[46]	2026	Journal paper	PHY	Complex indoor environments	Dynamic frequency hoping (DFH)	Real experiments and trace-driven simulation	PRR, energy consumption, throughput
[47]	2026	Preprint	PHY	DtS-IoT	Multi-domain instance fusion for interference recognition	Math. analysis, simulation	Exact accuracy, label accuracy, category-wise recall
[48]	2026	Conf. paper	MAC	Dense IoT networks	Probabilistic header repetition with SIC	Math. analysis, simulation	Average success delivery ratio, goodput
[52]	2024	Journal paper	PHY	DtS-IoT	Segment-level coding frequency hopping (SLC-FH)	Math. analysis, simulation	PLR, energy consumption

**Table 4:** References that include analytical models.

Category	Ref.	Year	Model
Link	[7]	2022	System, propagation/collisions, and capture effect
Link	[10]	2023	Outage probability in satellite channel
Link	[12]	2023	Simultaneous LR-FHSS signal reception with interference blanking
Link	[13]	2024	D2D-assisted communication in shadowed-Rice fading
Link	[14]	2023	LEO LR-FHSS transceiver and signal detection
Link	[18]	2025	Robust LR-FHSS synchronization and decoding model
Link	[19]	2024	GMSK-based PHY model
Link	[20]	2025	Enhanced Doppler-aware LR-FHSS receiver model
Link	[22]	2024	Message delivery probability model for LR-FHSS with message replication
Link	[23]	2025	Packet success probability and goodput model
Link	[26]	2024	Header and payload performance model for LR-FHSS in DtS-IoMT
Link	[28]	2025	Orthogonal allocation of physical channels for header and payload fragments
Link	[30]	2025	Urban scenario path-loss model
Link	[31]	2025	Optimal number of header replicas based on payload size and CR
Link	[34]	2025	Packet success probability in macro-diversity reception
Link	[37]	2025	ALOHA-based model of LR-FHSS packet success probability
Link	[39]	2025	SIC method for LR-FHSS to improve capacity
Link	[40]	2025	Three message-transmission strategies
Link	[41]	2026	Sensitivity-based outage for LEO/HAPS links
Link	[45]	2026	Packet loss rate under different interferers, for several header placement approaches
Link	[46]	2026	DFH PRR prediction model
Link	[52]	2024	Segment collisions, packet loss, and energy consumption
Link	[43]	2023	LoRa-CSS/LR-FHSS link-budget and airtime calculations
Link	[48]	2026	SIC method with probabilistic header repetition for LR-FHSS to improve capacity
Energy	[8]	2024	Energy-per-bit and battery-lifetime estimation model
Energy	[15]	2025	LR-FHSS ToA and current-consumption model
Energy	[27]	2024	Two-tier energy and throughput model for LR-FHSS
Energy	[40]	2025	Energy per message with the proposed transmission strategies
Other	[21]	2023	ILP model for headerless LR-FHSS frame recovery
Other	[42]	2026	2-D and sequential 1-D LoRa allocation benchmarks
Other	[47]	2026	Received-signal model and multi-instance multi-label recognition formulation

**Table 5:** References that include simulators.

Ref.	Year	Available	Simulator Description
[6]	2021	✓	Custom Matlab simulator for performance comparison of LoRa and LR-FHSS in terms of PRR
[7]	2022	✓	Custom Matlab simulator for PDR vs DR and payloads
[9]	2024	✓	LoRaSim-inspired energy efficiency simulator for LR-FHSS
[10]	2023	×	Custom computer simulation for outage probability vs number of nodes and node-satellite distances
[16]	2024	✓	(LR-FHSS-Sim) Models packet transmissions, interference, and network behavior, including ACRDA functionality
[18]	2025	×	Custom simulator for LR-FHSS over synthetic satellite channels
[19]	2024	×	Custom simulator for BER under different channel conditions
[20]	2025	✓	Python-based LR-FHSS collision simulator
[21]	2023	×	Custom Python-based LR-FHSS simulator with Gurobi solver for ILP
[22]	2024	×	Custom Matlab-based LR-FHSS replication simulator
[23]	2025	×	Custom discrete-event simulator in Python/SciPy
[26]	2024	×	Custom Matlab-based model for DtS-IoMT scenarios
[27]	2024	×	Custom Matlab simulator for topology-performance comparison
[28]	2025	×	Use the simulator proposed in [16] to evaluate the standard LR-FHSS and benchmark the results of their model
[31]	2025	×	Custom Python simulator modeling header and payload collisions to validate the analytical model
[33]	2025	×	Custom simulator for BER under different channel conditions
[35]	2025	×	Custom simulator based on Semtech’s drivers for LR11xx and SX126x devices
[36]	2025	×	Custom simulator for evaluating BER and ToA
[37]	2025	×	Use the simulator proposed in [16] as a ground truth to evaluate the results of their model and an alternative one
[38]	2025	✓	Custom simulator to assess the performance of the proposed FHSs and demodulation allocation strategies
[39]	2025	✓	Custom Matlab simulator to test a proposed SIC method
[40]	2025	×	Adapted simulator in [16] to test the performance of their proposals
[41]	2026	×	Monte Carlo outage evaluation for LEO/HAPS links
[42]	2026	×	Custom joint-access simulator using Skyfield/SGP4 orbital propagation
[45]	2026	×	Custom Monte Carlo simulator for PLR evaluation
[46]	2026	×	Custom-made trace-based simulation
[47]	2026	×	Synthetic US DR5 baseband dataset with fading, Doppler, and seven interference categories
[48]	2026	×	Adapted simulator in [16] to validate their analytical model

**Table 6:** List of references that include testbeds.

Ref.	Year	Details	Case Study
[8]	2024	ED, GW, and NS, all supporting LR-FHSS	Empirical measurements of ToA and current consumption of LoRaWAN-enabled LR-FHSS in the EU868 frequency band
[29]	2025	ED, GW, and NS, all supporting LR-FHSS	Experimental evaluation of LR-FHSS in urban and semi-urban scenarios
[15]	2025	ED supporting LR-FHSS	Empirical measurements of ToA and current consumption of LR-FHSS without LoRaWAN
[17]	2024	LR-FHSS transmitter and SDR-based LR-FHSS receiver	Evaluation of real-world LR-FHSS reception performance
[18]	2025	ASIC/FPGA-based transceiver	Verification of a custom LR-FHSS transceiver under realistic signal impairments
[24]	2024	Balloon and urban field test with LR-FHSS hardware	Real-world evaluation of LR-FHSS and LoRa under mobility and indoor conditions
[25]	2022	Semtech field and laboratory testing with SX1262 and a GW	Performance benchmarking of LR-FHSS and LoRa under realistic conditions
[30]	2025	ED, GW, and NS, all supporting LR-FHSS	Experimental evaluation of LR-FHSS in urban scenarios
[32]	2025	Balloon and urban field test with LR-FHSS hardware	Real-world evaluation of LR-FHSS and LoRa under mobility and indoor conditions
[44]	2026	ED and GW supporting LR-FHSS	Experimental evaluation in NLoS suburban metropolitan area
[46]	2026	Real transceivers and a software-defined GW	Experimental validation of DFH for LR-FHSS in interference scenarios
[43]	2023	Weather-station terminal and satellite reception	DEWASAT-1 case study: three-pass LoRa-CSS/LR-FHSS comparison

**Table 7:** References using reception, delivery, or error measures for performance evaluation. Each row indicates the packet, message, or network scope from its corresponding reference; metric definitions and comparison limits are given in Section 4.1.

Ref.	Year	Performance Metric	Highlighted Results
[7]	2022	Packet Delivery Success Probability	DR8 maintains a packet delivery success probability of ∼70% for 50,000 devices and outperforms DR9 under 150,000, but the latter becomes more reliable at higher counts
[25]	2022	PER	US DR5: 4% PER at 6.3 km, with 14 dBm transmit power in the measured suburban link
[9]	2024	Average Network Success	Average network success increases by up to 60% compared with default LR-FHSS in the studied scenarios
[10]	2023	Outage Probability	At a target outage probability of $10^{-2}$, US DR5 effectively doubles the GW capacity to 120,000 devices while extending the operational range to 1200 km
[11]	2024	Packet Error Probability	One order of magnitude decrease in PER
[12]	2023	PER	PER reduction in up to more than one order of magnitude
[14]	2023	PER	Achieves a PER lower than $10^{-3}$ in certain scenarios
[17]	2024	PRR	To maintain a 95% PRR across all channel types, the SNR must stay at or above −20 dB for DR8 and −16 dB for DR9
[18]	2025	PER	PER below $10^{-2}$ for DR9 and close to $10^{-4}$ for DR8
[19]	2024	BER	BER performance penalties relative to the optimal receiver: 1 dB in AWGN and 1.5 dB in shadowed-Rice fading
[22]	2024	Message Delivery Probability	3 message replicas yield a message delivery probability very close to 100% for 50,000 EDs using DR8
[24]	2024	Packet Delivery Success, PDR	While increasing transmit power can boost reliability for 30 km balloon-to-ground links, LR-FHSS is significantly more effective than traditional LoRa DRs in dense, short-range urban environments
[26]	2024	Probability of Successful Transmission	Packet transmission success probability beyond 0.9 and 0.7 for 50,000 and 100,000 EDs
[28]	2025	Probability of Successful Transmission	Higher reliability than standard LR-FHSS in all scenarios
[29]	2025	PDR	Up to ∼90% PDR for a distance between ED and GW of 4 km
[30]	2025	PRR	At 1.5 km: 85% PRR for US DR5 versus 75% for US DR6 and US LoRa DR0, with 22 dBm transmit power and a 4-byte payload
[32]	2025	PDR	Apart from results in [24], LR-FHSS is more sensitive than LoRa to the CR being used
[33]	2025	BER	At a $10^{-4}$ BER in AWGN, the proposed filter’s performance degradation against interference-free conditions is just 2 dB, 1 dB, and <0.5 dB for SFs 7, 9, and 11
[34]	2025	Packet Success Probability	At 80% packet success probability, sustainable offered load per GW increases from 1.7 to 7.9 Mbps for US DR5 and from 0.63 to 4.2 Mbps for US DR6
[35]	2025	Miss detection probability	Good header detection performance for Doppler rates up to 250 Hz/s
[37]	2025	Packet Success Rate (probability)	More accurate performance prediction in a wider range of scenarios than other models
[40]	2025	Message Success Probability	Concatenation achieves up to 44% higher message success probability than regular LR-FHSS at 50,000 EDs
[41]	2026	Outage probability	LR-FHSS and SF12 share modeled outage; HAPS permits lower SFs
[42]	2026	PDR	Higher PDR above roughly 20–25 packets/s with two LoRa channels and one LR-FHSS channel, under favorable visibility
[44]	2026	PRR	Up to 15% PRR increase for confirmed traffic, by extending the link range of LR-FHSS signals repurposed as LoRa ACKs and enabling retries for uplink-only commercial devices.
[45]	2026	PLR	Minor PLR decrease for one of the modified header placement approaches considered.
[46]	2026	PRR	Increases PRR by up to a factor of 2.76
[52]	2024	PLR	Segment-level erasure coding improves resilience to clustered errors
[43]	2023	Received packet count	LR-FHSS receives more packets in two of three reported passes

**Table 8:** List of references using performance metrics related to energy.

Ref.	Year	Performance Metric	Highlighted Results
[8]	2024	Average current consumption, battery lifetime, and energy efficiency	With a 230 mAh battery and a 50 min transmission interval, LR-FHSS devices achieve a lifetime of 1.2 to 2.5 years, comparable to LoRa DR0
[15]	2025	Current consumption and battery lifetime	For a 2400 mAh battery, 10-byte payload, 30 min interval, and 14 dBm, modeled LR-FHSS lifetime exceeds 4.2 years; DR9 gives the lowest energy per successfully delivered packet in the evaluated scenarios
[22]	2024	Energy efficiency	There is a trade-off between signal robustness and energy efficiency
[23]	2025	Energy efficiency	Optimizing the configuration probabilities improves energy efficiency over standard DR8/DR9 in the evaluated scenarios; its optimum differs from the goodput optimum
[25]	2022	Annual electric charge consumption (in the document, annual power consumption)	323 mAh (CR 1/3) and 258 mAh (CR 2/3), for six reports per day with a 20-byte PHY payload; LoRa SF10/SF12: 205/280 mAh without repetitions
[27]	2024	Energy consumption	A two-tier network using LR-FHSS is the most energy-efficient option
[40]	2025	Energy per message	The concatenation technique extends battery lifetime by reducing protocol overhead
[42]	2026	Relative transmission energy	ALOHA-based joint policies use about 80% of pure LR-FHSS energy near 20 packets/s under favorable visibility

**Table 9:** List of references using the number of supported devices as a performance metric.

Ref.	Year	Highlighted Results
[9]	2024	Up to tens of thousands of devices per GW
[10]	2023	Up to 120,000 EDs per hour for 48-byte payloads at a target outage probability of $10^{-2}$
[12]	2023	A 2.5-fold increase in the capacity to decode LR-FHSS signals
[13]	2024	Capacity increases of 249.9% for US DR6 and 150.1% for US DR5 at outage probability $10^{-2}$
[17]	2024	Confirms that the protocol meets its high-capacity expectations
[28]	2025	Supports up to 50% more clients at the same reliability as standard LR-FHSS
[31]	2025	Over 18,000 EDs per GW with aligned subchannels, DR8, 10-byte payloads, and maximum-capacity traffic
[38]	2025	Network decoding capacity can be increased by as much as 100% in specific configurations
[40]	2025	The concatenation technique alleviates network congestion by reducing the number of transmissions competing for the channel

**Table 10:** List of references using throughput or goodput as performance metrics.

Ref.	Year	Performance Metric	Highlighted Results
[6]	2021	Throughput	Measures the raw protocol capabilities
[12]	2023	Throughput	Enhances throughput through improved FHS detection
[16]	2024	Throughput	Provides a simulation tool for evaluating throughput
[27]	2024	Throughput	Improves throughput through the use of relay nodes
[9]	2024	Goodput	Interference cancellation increases goodput, particularly in saturated scenarios
[23]	2025	Goodput	Maximizes goodput through dynamic configuration adjustments
[25]	2022	Goodput	Assesses the raw capabilities of the technology
[31]	2025	Goodput	Predicts a threefold goodput improvement over the earlier simulation results under its modeling assumptions
[34]	2025	Goodput	Macro-diversity achieves up to a fivefold improvement in peak goodput
[39]	2025	Goodput	Roughly doubles network capacity at 90% PRR using a robust SIC method
[42]	2026	Throughput	Unique payload per window trades off against PDR as access thresholds change
[48]	2026	Goodput	Performance is maximized using a probabilistic header repetition scheme combined with a SIC mechanism

**Table 11:** List of references using latency or ToA for performance evaluation.

Ref.	Year	Performance Metric	Highlighted Results
[8]	2024	ToA	Quantifies that LR-FHSS generally presents a higher ToA than LoRa
[15]	2025	ToA	Quantifies that LR-FHSS generally presents a higher ToA than LoRa
[25]	2022	Latency	States that the higher reliability of LR-FHSS provides more stable latency
[26]	2024	ToA	Proposes omitting the header transmission to reduce ToA
[40]	2025	Latency	Replication offers the lowest average delay, whereas concatenation produces the highest delays
[43]	2023	ToA	1.868/1.051 s for CR 1/3 and 2/3, respectively; 20-byte payload with CRC

**Table 12:** Cross-reference of LR-FHSS improvement techniques, evaluation methods, reported outcomes, and target applications. Metric definitions and reception units are distinguished in Section 4.1.

Technique and Reference	Evaluation Method	Reported Outcome/Trade-Off	Target Application/Context
Headerless frame recovery [20,21]	ILP; Python simulations, with Gurobi for the ILP	Greedy recovery is less time-consuming than ILP; the Doppler-aware extension reports up to 50% higher PDR in simulated terrestrial/satellite scenarios.	DtS-IoT; terrestrial scenarios also simulated (Section 6.1).
Low-complexity GMSK detection [19]	PHY analysis; AWGN and shadowed-Rice simulations	Lower detection complexity; 1 dB and 1.5 dB BER performance penalties in AWGN and shadowed-Rice fading, respectively.	DtS-IoT (Section 6.1).
Synchronization and transceiver design [14,18]	Analysis; simulation; ASIC/FPGA tests in the later work	Later tests: PER below $10^{-2}$ for DR9 and approaching $10^{-4}$ for DR8, with eight transmitters.	DtS-IoT (Section 6.1).
Two-stage header detection [35]	Simulation using Semtech drivers	Discrete Fourier transform size more than ten times smaller than the reference implementation; evaluated at Doppler rates up to 250 Hz/s.	DtS-IoT (Section 6.1).
Interference blanking [12]	Analysis and simulation	PER reduced by over one order of magnitude in the evaluated scenarios.	DtS-IoT (Section 6.1).
SIC and IPAD [17]	SDR measurements; trace-driven simulation	PRR improved by up to an additional ∼15% at high traffic loads.	DtS-IoT; terrestrial and NTN channel evaluation (Section 6.1).
Robust SIC with multi-symbol averaging [39]	Analysis; MATLAB trace-driven ETU simulation	Approximately 7.9/7.4 kbps for DR8/DR9 at 90% PRR in 137 kHz.	Terrestrial channel evaluation; DtS also discussed (Section 6.2).
ACRDA integration [9]	Analysis; LoRaSim-inspired simulation	Up to 60% higher average network success and approximately 100% higher goodput in the studied scenarios.	DtS-IoT (Section 6.1).
Fragment-level macro-diversity [34]	Analytical modeling	Up to fivefold peak goodput versus nearest-GW reception.	Large-scale, multi-GW IoT (Section 6.3).
DFH [46]	Analysis; Monte Carlo simulation; indoor SDR testbed; trace-driven simulation	Up to 2.76× PRR versus standard LR-FHSS.	Terrestrial indoor IoT (Section 6.2).
Frame and fragment replication [22]	Analysis; MATLAB Monte Carlo simulation	Frame replication favors low traffic; fragment replication favors high traffic. Reliability gains incur an energy cost.	DtS-IoT (Section 6.1).
Orthogonal header/payload channel allocation [28]	Analysis; LR-FHSS-Sim	Up to 50% more clients at the same reliability as standard LR-FHSS.	Dense IoT; DtS-IoT (Section 6.1).
Wide-Gap FHS; Early-Decode/Early-Drop; header tolerance [38]	Custom simulation for DR8/DR9	Up to 50% higher decoding capacity for combined enhancements; results depend on configuration.	DtS-IoT (Section 6.1).
Concatenation, embedded retransmission, replication [40]	Analysis; adapted LR-FHSS-Sim	Concatenation: up to 44% higher message success probability at 50,000 EDs, at higher delay; embedded retransmission favors lower densities.	DtS-IoT; delay-tolerant reporting (Section 6.1).
Network-coded header replication [11]	Analysis and simulation	PER reduced by more than one order of magnitude for DR8.	Terrestrial IoT (Section 6.2).
Probabilistic CR/header-replica allocation [23]	Analysis; Python/SciPy discrete-event simulation	Goodput and energy-efficiency optima require different configuration distributions.	Dense sensor networks (Section 6.3).
Header redundancy optimization [31]	Analysis; discrete-event simulation	At CR 1/3, four headers improve utilization for 72% of packets in the considered CampusIoT dataset.	Dense IoT; DtS-IoT (Section 6.1).
D2D-aided satellite access [13]	Analysis and simulation	Capacity gains of 249.9% (US DR6) and 150.1% (US DR5) at outage probability $10^{-2}$.	Satellite IoT with relays (Section 6.1).
Two-tier LoRa/LR-FHSS relaying [27]	Analysis; MATLAB simulation	Energy savings with a relayed two-tier architecture.	Large-scale IoT with satellite backhaul (Section 6.3).
Header omission [26]	Analytical models; MATLAB simulation	Proposed to reduce ToA; a separate numerical gain for header omission is not given in this survey.	Satellite IoMT monitoring (Section 6.3).
Filtering-based LoRa detection under LR-FHSS interference [33]	AWGN and Rayleigh fading simulations	At BER $10^{-4}$ in AWGN: 2, 1 and <0.5 dB penalties versus interference-free conditions for SF7, SF9, and SF11.	Terrestrial LoRa/DtS coexistence (Section 6.2).
Using LR-FHSS signals repurposed as LoRa downlink signals [44]	Experimental	Up to 15% PRR increase for confirmed traffic.	DtS-IoT and terrestrial (Section 6.1 and Section 6.2).
Header repetition placement modification [45]	Math. analysis, simulation	Packet Loss Rate decrease for one of the modified header placement approaches considered.	DtS-IoT (Section 6.1).
SLC-FH over high-order Galois fields with erasure detection [52]	Math. analysis and simulation	10× more users at $10^{-3}$ packet loss rate, and 50% lower energy consumption than a baseline.	DtS-IoT (Section 6.1).
Joint LoRa/LR-FHSS access [42]	Optimization; constellation simulation	Visibility/load-aware access balances PDR, unique throughput, and transmission energy.	DtS-IoT (Section 6.1).
Compound interference recognition [47]	Synthetic-signal simulation; learning-based classification	36.36% overall exact accuracy versus 21.65% for the strongest baseline, using clean/single-interferer training samples.	DtS-IoT receiver monitoring (Section 6.1).

## Data Availability

No new data were created or analyzed in this study. Data sharing is not applicable to this article.
